# Indole-Containing Phytoalexin-Based Bioisosteres as Antifungals: In Vitro and In Silico Evaluation against *Fusarium oxysporum*

**DOI:** 10.3390/molecules25010045

**Published:** 2019-12-21

**Authors:** Andrea Angarita-Rodríguez, Diego Quiroga, Ericsson Coy-Barrera

**Affiliations:** Bioorganic Chemistry Laboratory, Facultad de Ciencias Básicas y Aplicadas, Universidad Militar Nueva Granada, Campus Nueva Granada, Cajicá 250247, Colombia; u0500924@unimilitar.edu.co (A.A.-R.); diego.quiroga@unimilitar.edu.co (D.Q.)

**Keywords:** phytopathogens, *Fusarium* spp., antifungals, indole-containing analogs

## Abstract

There is a continuous search for more reliable and effective alternatives to control phytopathogens through different strategies. In this context, indole-containing phytoalexins are stimuli-induced compounds implicated in plant defense against plant pathogens. However, phytoalexins’ efficacy have been limited by fungal detoxifying mechanisms, thus, the research on bioisosteres-based analogs can be a friendly alternative regarding the control of *Fusarium* phytopathogens, but there are currently few studies on it. Thus, as part of our research on antifungal agents, a set of 21 synthetic indole-containing phytoalexin analogs were evaluated as inhibitors against the phyopathogen *Fusarium oxysporum*. Results indicated that analogs of the *N*,*N*-dialkylthiourea, *N,S*-dialkyldithiocarbamate and substituted-1,3-thiazolidin-5-one groups exhibited the best docking scores and interaction profiles within the active site of *Fusarium* spp. enzymes. Vina scores exhibited correlation with experimental mycelial growth inhibition using supervised statistics, and this antifungal dataset correlated with molecular interaction fields after CoMFA. Compound **24** (*tert*-butyl (((3-oxo-1,3-diphenylpropyl)thio)carbonothioyl)-l-tryptophanate), a very active analog against *F. oxysporum*, exhibited the best interaction with lanosterol 14α-demethylase according to molecular docking, molecular dynamics and molecular mechanic/poisson-boltzmann surface area (MM/PBSA) binding energy performance. After data analyses, information on mycelial growth inhibitors, structural requirements and putative enzyme targets may be used in further antifungal development based on phytoalexin analogs for controlling phytopathogens.

## 1. Introduction

Fungi from the genus *Fusarium* are very important because they have wide cosmopolitan-type geographic distribution and high relevance for agriculture and economy [1]. Their diversity is quite common in soils and many of them have the capacity to cause infectious diseases in different types of crop plants. Additionally, some species can cause opportunistic infections in animals due to the production of toxins that can affect them [1]. Genus *Fusarium* gathered phytopathogenic-behaved species causing necrosis-associated diseases in several plants around the world. This fungus can survive in the soil in mycelial or conidial forms during host absence. When a host is present, the mycelium penetrates its roots, enters the vascular system (xylem) to move and multiply itself, causing wilt symptoms. Soil-borne fungi *Fusarium* spp. can infects the host by using different sets of genes for early host-plant signaling, as well as the binding and enzymatic decomposition of antifungal compounds produced by plant for defense, in order to inactivate and kill host cells by fungal toxins [2]. In this context, some control strategies based on chemical, physical and cultural methods have arisen to mitigate the negative effects of this phytopathogen to host plants. Chemical treatments include formaldehyde applications to avoid disseminations as well as dazomet, sodium methan, methyl isothiocyanate and systemic fungicides as benomyl, thiabendazon, carbendazim and methylthiophanate [3]. *Fusarium*-caused diseases treatment by systemic fungicides, although it has good effectivity, is also problematic, since these kind of treatments can become mutagenics for several plants and these agents can also generate high degrees of resistance [3,4].

Plants involve a systemic defense mechanism involving some metabolites such as phytoalexins. These metabolites are synthesized in adjacent areas of healthy cells to those damaged cells and they are accumulated both in necrotic and susceptible resistant tissues [5]. In other words, they are strictly produced in a site around the place where infection needs to be controlled. Thus, the resistance occurs when one or more phytoalexins reach a high enough concentration to inhibit the pathogen development [5]. However, some phytopathogens are recently described to exhibit strategies to invade the plant tissues and obtain necessary nutrients for its growing and reproduction [1,2,4]. Despite that plants are capable to synthesize specific antifungal metabolites, the fungi are capable to produce particular enzymes in order to detoxify quickly and inhibit the phytoalexin activity. This detoxifying capacity is described by Pedras et al. [6] as an “arms race” that advantaged the pathogen, causing adverse effects in the crop. An example of the previous fact is the case of brassinin (an important phytoalexin for crucifers due its dual potential as an antimicrobial defense and a biosynthetic precursor of phytoalexin variety), since if this metabolite is inhibited by a fungal enzyme, the plant become possibly more susceptible to be attacked by the phytopathogen [6]. Thus, the cruciferous invader *L. maculans* (a devastating pathogen) can generate itself a detoxification defense against phytoalexins. The mechanism is based on brassinin oxidase production, which catalyzes the brassinin oxidation through the dithiocarbamate transformation into an aldehyde. This mechanism depends on direct brassinin recognition by this enzyme. Therefore, exploration of brassinin analogs acting as bioisosteres can be adopted as an important approach for the development of novel antifungals [7]. In this sense, bioisosterism is generally a chemical strategy for the rational design of bioactives from structural modifications of leads, whose action mechanism and chemical structure is therefore known. From the above fact, it is possible to develop novel compounds with chemical and/or physical similarities but producing identical or even better biological properties [7]. Therefore, as part of our research on antifungals discovery and development, the present work was initially focused on the in silico exploration, by molecular docking, on the interaction of a set of indole-containing phytoalexin analogs within the active site of 25 enzymes reported to *Fusarium* spp. Subsequently, the integration of the experimental antifungal activity (i.e., *Fusarium oxysporum* mycelial growth inhibition) and docking scores datasets (by supervised multivariate statistics) as well as the three-dimensional (3D) molecular descriptors and antifungal activity datasets (on the basis of 3D quantitative structure-activity relationship (3D-QSAR) by comparative molecular field analysis (CoMFA)) led to recognize some important hits, enzyme targets and structural requirements to be stated for the further development of antifungals for controlling phytopathogens using phytoalexin-like analogs. Finally, some insights into the binding mode of a 3-oxo-1,3-diphenylpropyl *N*-alkyldithiocarbamate-type analog and the homology model of the transmembrane *F. cerealis* lanosterol 14-demethylase were also determined in order to rationalize the antifungal activity of this type of synthetic phytoalexin analogs.

## 2. Results and Discussion

### 2.1. Test Indole-Containing Phytoalexin Analogs and Docking Protocol Validation

**Figure 1:**
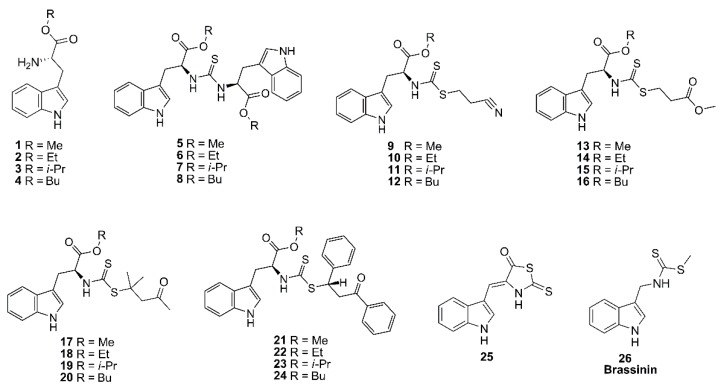
Structural classification of the test indole-containing phytoalexin analogs: 2-alkyl aminoesters (**1**–**4**), *N*,*N*-dialkylthioureas (**5**–**8**), 2-cyanoethyl *N*-alkyldithiocarbamates (**9**–**12**), 3-methoxy-3-oxopropyl *N*-alkyldithiocarbamate (**13**–**16**), 2-methyl-4-oxopentan-2-yl *N*-alkyldithiocarbamate (**17**–**20**), 2-oxo-1,3-diphenylpropyl *N*-alkyldithiocarbamates (**21**–**24**), 4-[(1*H*-indol-3-yl) methylene]-2-sulfanilidene-1,3-thiazolidin-5-one (**25**), brassinin (**26**).

A set of 25 synthetic indole-containing compounds (**1**–**25**) (Figure 1) were obtained by our previously reported reaction route from l-tryptophan [8] (Figure S1). Their IUPAC names are displayed in the complementary material (Table S1). Such compounds were then chosen as test compounds owing to their antifungal activity by inhibiting mycelial growth of *F. oxysporum* [8,9]. Thus, in order to explore and outline a further information related to the potential and putative targets of these compounds at in silico level, docking simulations were therefore performed with 25 fungal enzymes (E1–E25) (Table A1 (Appendix A)), which were selected due to their significant associations on pathogenic and metabolic performance of *Fusarium* spp. 

**Figure 2:**
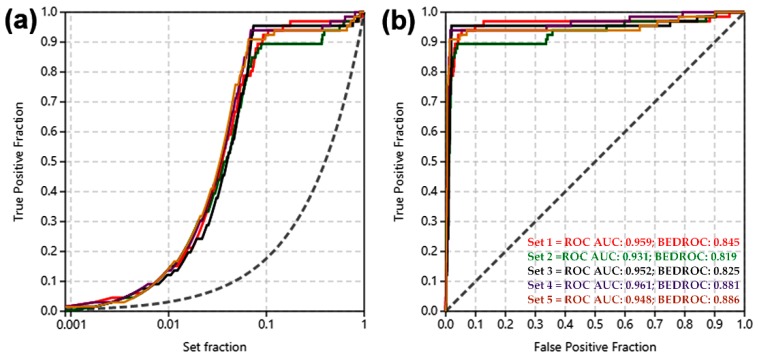
Benchmarking of the docking protocol using a set of active compounds decoys against test enzymes (red line = isocitrate lyase; green line = aspartate kinase; black line = serine esterase; purple line = lanosterol 14α-demethylase; dark orange line = trichothecene acetyltransferase). (**a**) Receiver operating characteristic (ROC). (**b**) Enrichment curve.

Among the several in silico approaches for the study of active chemical agents from public or in-house libraries [10], molecular docking was then selected due to the capacity to simulate the binding mode of low-molecular weight compounds within the active site of enzymes and binding pocket of receptors. This is a very important procedure for the prediction of putative targets, as the first-line step in the search for plausible mechanism of action of active compounds [11]. However, some problems/issues can arise and they should be taken into account for caution and concern during structure-based screening [12], as well as the selection of suitable protocol for sampling and scoring procedure to discriminate potential binders from non-binders using accurate parameters [13]. In this regard, the molecular docking of the co-crystalized ligands (CCL) for each enzyme let us evaluate the good performance of the docking protocol used, since this re-docking calculations resulted into conformational root-mean-square deviation (RMSD) values < 1.0, indicating that our parameters were suitable for docking. Additionally, this performance was also evaluated through a benchmarking strategy using a set of decoys (*n* = 1125) and active compounds (*n* = 55) against five enzymes within test set (i.e., isocitrate lyase, aspartate kinase, sereine esterase, lanosterol 14α-demethylase and trichothecene acetyltransferase) having known inhibitors, retrieved from literature and databases. The sensitivity and specificity of the docking protocol was assessed by calculation of the area under curve (AUC) from receiver operating characteristic (ROC) curves [14]. This assessment of our docking protocol reached true positive identification over 90% of the active compounds involving a recognition within 10% of the test compounds (Figure 2a). Therefore, the validation of the docking protocol was considered successful, since the AUC and the Boltzmann-enhanced discrimination of the receiver operating characteristic (BEDROC) fell into 0.931–0.961 and 0.819–0.886 ranges, respectively (Figure 2b). Previously, such a kind of validation let to the discovery of new chemotypes for inhibition of homoserine dehydrogenase [15], as an indication of the importance of validating the docking protocols.

Once the parameters were adequately validated, each Merck molecular force field (MMFF)-optimized structure **1**–**26** was docked into the active site of each enzyme using AutoDock/Vina. The calculated affinities (as Vina scores, expressed in kcal/mol) of the resulting enzyme-ligand complexes, for each enzyme and compound, were compiled as a data matrix summarized in Table S2. This dataset revealed some trends around the affinity values as a result of the simulated molecular interaction between analogs and enzyme targets, through the use of descriptive and multivariate analysis. For this purpose, test compounds **1**–**25** were subdivided into seven classes according to their moieties: alkyl 2-aminoesters (AAE) **1**–**4**, *N*,*N*-dialkylthioureas (NNDATU) **5**–**8**, 2-cyanoethyl *N*-alkyldithiocarbamates (CE-DTC) **9**–**12**, 3-methoxy-3-oxopropyl *N*-alkyldithiocarbamate (MOPr-DTC) **13**–**16**, 2-methyl-4-oxopentan-2-yl *N*-alkyldithiocarbamate (MOPe-DTC) **17**–**20**, 2-oxo-1,3-diphenylpropyl *N*-alkyldithiocarbamates (ODP-DTC) **21**–**24** and 4-[(1*H*-indol-3-yl)-methylene]-2-sulfanylidene-1,3-thiazolidin-5-one (IST) **25**, along with brassinin (**26**) as the reference phytoalexin [6].

### 2.2. Vina Scores-Related Trends

The affinity values for each ligand-enzyme complexes were compiled into a full data matrix. A reduced dataset expressed as mean Vina scores ± standard deviation (SD)—after 10 independent calculations—is exposed in Table S2. The SD for Vina scores exhibited good performance between replicates, having coefficient of variation into the 5–15% range, although with some outliers. The root-mean-square deviation (RMSD) of atomic positions for the best-ranked poses between replicates exhibited excellent convergence (<1.0 Å). The lowest scores per enzyme and ligand were highlighted in red. From these data, ODP-DTC, NNDATU and IST-type ligands were found to generate more stable ligand-enzyme complexes with 13, eight and four enzymes, respectively. These resulting Vina scores were analyzed by Pearson’s correlation (Table 1). Thus, the respective coefficients were independently calculated between test compounds as well as among docked enzymes. Firstly, the Pearson’s coefficients (Pc) for some pair of compound classes, according to their complete dataset of Vina scores for all enzymes, exhibited values above 0.9. This correlation indicated a similar interaction profile with the enzyme group.

Therefore, AAE exhibited similar performance with MOPe-DTC and IST, whereas MOPr-DTC with AAE, CE-DTC, MOPe-DTC and IST. NNDATU and ODP-DTC exhibited coefficient values below 0.75 and 0.65, respectively, suggesting different trends in the molecular docking results in comparison to the other compound classes. Regarding the Pc across enzymes (Table S3), 13 paired correlations were detected with values above 0.8, which implies similar interaction profile according to the structural variations of test compound set and therefore depending possibly by the size, shape and/or chemical behavior of the active site of each enzyme and even enzyme family. For instance, the pairs E2–E3 and E18–E19 were found to be correlated (Pc = 0.879 and 0.812, respectively) and they belong to the same enzyme families, i.e., glycosyl hydrolase and nitroalkane oxidase families, respectively. This outcome suggested good performance of the docking calculations in accordance with the good convergence found in the output data. Additionally, E5 showed good correlation with E2 and E3 (Pc > 0.75), but E5 is part of a different enzyme family, i.e., transferase that catalyzes the transfer of an acetyl group to the hydroxyl at C3 of several trichothecene-type mycotoxins [16]. In this sense, it is reasonable to consider that such a tendency is owing to the presence of similar docking interactions between residues from active site of different enzymes and the compound set. In Table S3 are highlighted those correlated enzyme pairs from resulting Vina scores after docking that support these observations.

**Figure 3:**
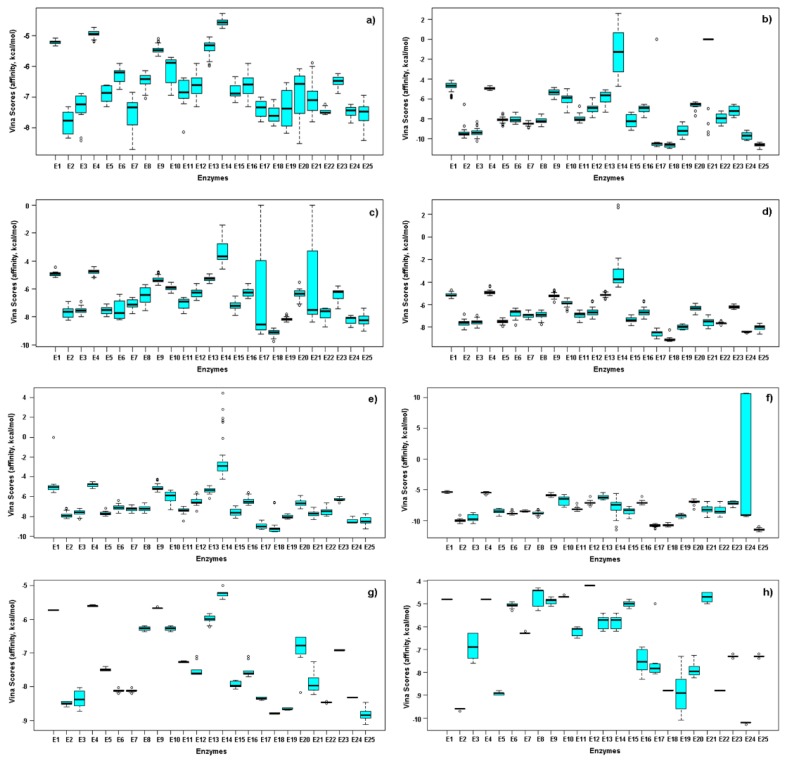
Boxplots for affinity values (as Vina scores in kcal/mol) after molecular docking for test phytoalexin analogs. (**a**) 2-alkyl aminoesters (AAE), (**b**) *N*,*N*-dialkylthioureas (NNDATU), (**c**) 2-cyanoethyl *N*-alkyldithiocarbamates (CE-DTC), (**d**) 3-methoxy-3-oxopropyl *N*-alkyldithiocarbamate (MOPr-DTC), (**e**) 2-methyl-4-oxopentan-2-yl *N*-alkyldithiocarbamate (MOPe-DTC), (**f**) 2-oxo-1,3-diphenylpropyl *N*-alkyldithiocarbamates (ODP-DTC), (**g**) 4-[(1*H*-indol-3-yl) methylene]-2-sulfanilidene-1,3-thiazolidin-5-one (IST), (**h**) brassinin.

In order to observe the particular variation/distribution of Vina scores between enzymes and test ligands, the respective box plots were also constructed using the whole dataset (Figure 3). Thus, 12 enzymes exhibited good reproducibility and low dispersion in the docking calculations for six types of ligands, whereas AAE and CE-DTC showed more data scattering. 

The best behavior was exhibited by ODP-DTC-type phytoalexin, excepting for E24, because two outliers were produced for **21** and **22**. A similar dispersion was also observed for other compound type on interacting with specific enzymes, indicating precise structural requirements to interact depending on the alkyl group at ester moiety. Concerning the best-docked complexes, MOPe-DTC and CE-DTC-type ligands were associated to a best interaction with E18, whereas MOPr-DTC-type analogs might inhibit putatively the enzymes E18 and E24. ODP-DTC-type compounds showed the best Vina scores with E2, E17, E18 and E25. Regarding brassinin, its behavior was found to be comparable to that of IST. These facts led to infer that significant differences in the structural interactions between enzymes and test ligands would influence the capacity to form a stable complex.

### 2.3. Unsupervised and Supervised Multivariate Statistics 

**Figure 4:**
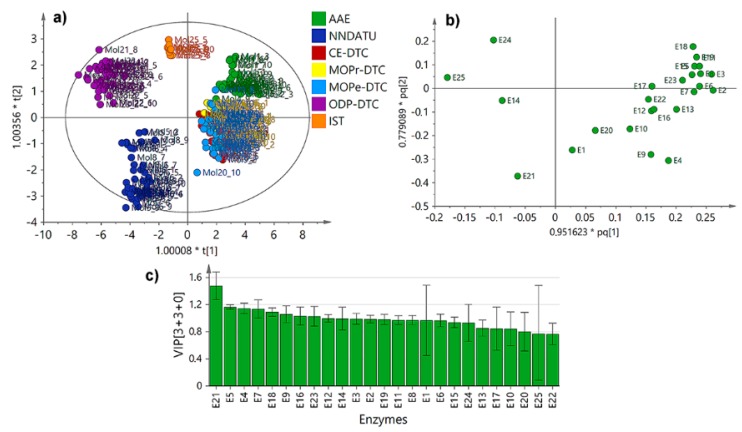
Supervised multivariate analysis from Vina scores for indole-containing phytoalexin analogs. (**a**) Orthogonal partial least squares – discriminant analysis (OPLS-DA)-derived score plot. Coloration according compound type: alkyl 2-aminoesters (AAE), *N*,*N*-dialkylthioureas (NNDATU), 2-cyanoethyl *N*-alkyldithiocarbamates (CE-DTC), 3-methoxy-3-oxopropyl *N*-alkyldithiocarbamate (MOPr-DTC), 2-methyl-4-oxopentan-2-yl *N*-alkyldithiocarbamate (MOPe-DTC), 2-oxo-1,3-diphenylpropyl *N*-alkyldithiocarbamates (ODP-DTC), 4-[(1*H*-indol-3-yl)-methylene]-2-sulfanylidene-1,3-thiazolidin-5-one (IST); (**b**) OPLS-DA-derived loadings plot; (**c**) Variable importance in projection (VIP) scores plot. X-scores (t[1] or t[2]) in **(a)** and XY-combined loadings (pq[1] or pq[2]) in **(b)** were proportionally scaled to the explained variation (R^2^_X_) using a coefficient as multiplicative factor (*).

The plausible relationships, from a holistic point-of-view, were studied through multivariate analysis using the Vina scores dataset for each enzyme (as variables) and test compounds/natural ligands (as observations). Hence, an unsupervised exploration through principal component analysis (PCA) was firstly started and the resulting PC1 versus PC2 was obtained (Figure S2). As expected, the docking results of natural ligands (NL) showed a different behavior to that of those Vina scores of all test analogs (Figure S2a); thus, they were excluded in order to start an additional PCA. Figure S2b,c show the resulting score plot (uncolored and colored by compound type, respectively), which exhibits a clustering depending on the different classes of compounds within dataset. This examination was then refined using a supervised examination by means of Orthogonal Partial Least Squares-Discriminant Analysis (OPLS-DA) using the compound type as categorical variable, in order to observe the particular connection of the docking scores related to the structures of test analogs. In summary, the OPLS-DA-derived score plot defined more clearly that NNDATU, IST and ODP-DTC were clustered as independent groups in comparison to the other compound type (Figure 4a) as evidenced in Pearson correlations and box plots distributions. 

The respective loadings plot (Figure 4b) exposed those variables (i.e., enzymes) that influence the observations (i.e., test analogs) discriminations across the Vina scores. In this regard, NNDATU, IST and ODP-DTC were reasonably influenced by the resulting docking scores with the E14, E21, E24 and E25, which are related to the lyase, dehydrogenase, and oxidoreductase enzyme families, respectively. The influence contribution of variables across dataset within the OPLS-DA model [3+3+0] was estimated by the variable importance in projection (VIP) scores. E25 showed the most deviated VIP score, indicating probable good selectivity on the contribution particularly due to lowest docking scores for some specific compound types (such as ODP-DTC), whereas E21 exhibited the highest one, i.e., poorest selectivity by some compound dataset (such as NNDATU). Particularly, NNDATU possessed several chemical and biological properties that are directly related to their molecular structure. Their observed differences are due to their substitutions and such specific conformations they can adopt around the thioamide moiety [17]. 

**Figure 5:**
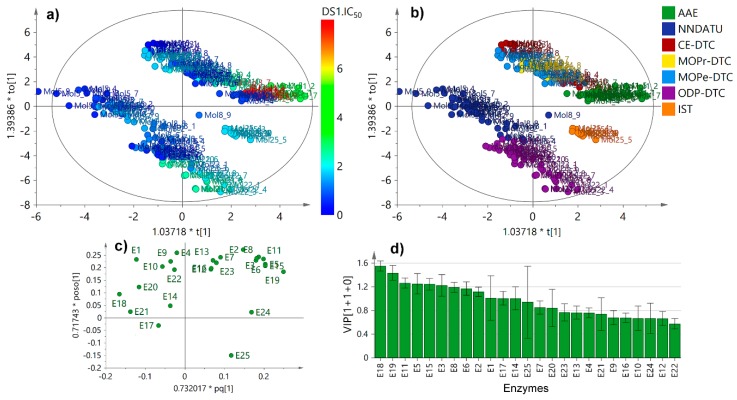
Supervised multivariate analysis using Single-*Y* OPLS on Vina scores data (as X) and antifungal activity (half-maximal inhibitory concentrations (IC_50_ in mM) as Y). (**a**) Single-Y OPLS-derived score plot. Coloration according heatmap of IC_50_ dataset correlation; (**b**) Single-Y OPLS-derived score plot. Coloration by compound type; (**c**) Single-Y OPLS-derived loadings plot; (**d**) Variable importance in projection (VIP) scores plot. X and orthogonal scores (t[1] or to[1], respectively) in (**a**,**b**) and XY-combined and orthogonal loadings (pq[1] or poso[1], respectively) in (**c**) were proportionally scaled to the explained variation (R^2^_X_) using a coefficient as multiplicative factor (*).

In order to estimate such tendencies experimentally and compare them with the performance of the present docking study, the mycelial growth inhibition was evaluated for all compound set against *F. oxysporum*. Such evaluation was built on our prior study to expand the search for promising antifungal agents [8] and the resulting half-maximal inhibitory concentrations (IC_50_) are exposed in Table S4. Test compounds exhibited mycelial growth inhibition at different levels, but some NNDATU, OPD-DTC and CE-DTC exhibited the lowest IC_50_ values (<0.8 mM), thus, they can be considered as the best antifungals within the experimental dataset. A clear structure-activity relationship was not noticeably evidenced; thus, the antifungal data was then used for supervised analysis in order to integrate the docking and biological activity datasets. Hnece, the supervised analysis for docking scores by single-*Y* OPLS regression, using the experimental IC_50_ values as continuous variables (i.e., Y) (Figure 5), clustered similarly the observations into some groups according to the compound type under the corresponding regression (Figure 5a,b). 

Once again, the well-defined, separated groups were NNDATU, OPD-DTC and IST. These results can rationalized since NNDATU and OPD-DTC exhibited similar docking profile between them, whereas IST was slightly different; however, these three kind of compounds were very distinct from the others. This fact represented a clear trend between docking scores performance and the experimental IC_50_ values based on compound type, justifying conceivable structure-activity and structure-affinity relationships. Furthermore, the differential variables (i.e., enzymes) were identified through the loadings. The respective loadings plot (Figure 5c) showed a remarkable influence of E14, E17, E18, E20 and E21 for the separation of NNDATU-based clusters, while IST and OPD-DTC were most influenced by E24 and E25, respectively. Therefore, these results are in accordance with the respective box-plots (Figure 2) using only the Vina scores performance for each compound type. Twelve enzymes revealed VIP scores ≥ 1.0 under IC_50_ supervision, thus, they can be considered important in the given model to integrate in silico docking scores and in-vitro antifungal activity (Figure 5d). In this regard, E18 was the most influencing variable, and E25 persisted as the most deviated influencing enzyme. This last fact is an indicative of a particular selectivity of some compounds (i.e., OPD-DTC-type) to interact with this enzyme (E25).

*N*,*S*-dialkyl dithiocarbamates have already been described as potential fungicides, specifically as mycelial growth inhibitors of several pathogenic fungi [9,18], but *N*-alkyl dithiocarbamate salts are largely commercialized as fungicides, since they have multi-site action [19]. In general, dithiocarbamates become toxic when they are metabolized by the target fungus, especially on producing the isothiocyanate radical. It can be captured, e.g., by sulfhydryl groups in amino acids and enzymes; thus, the enzymatic activity is inhibited together with subsequent biological disruptions, such as lipid metabolism, cell membrane permeability and respiration and ATP production, among others [20]. 

### 2.4. Comparative Molecular Field Analysis (CoMFA)

**Figure 6:**
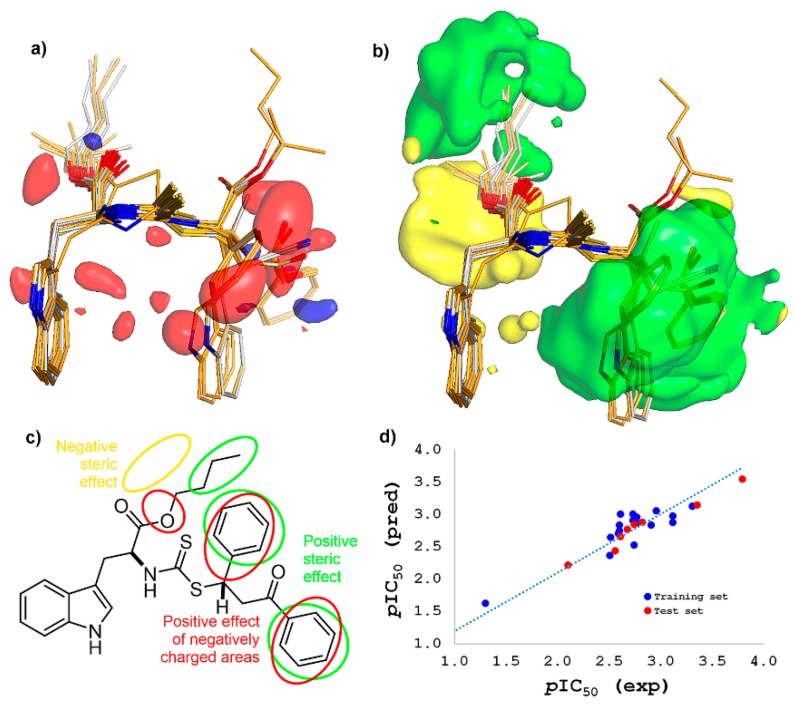
Comparative molecular field analysis (CoMFA). Steric (**a**) and Electrostatic (**b**) maps after the CoMFA model. Green and yellow contours expose positions where a bulky group would be favorable and unfavorable, respectively. Blue and red contours comprise regions where an increase of positive and negative charges, respectively, will enhance the antifungal activity; (**c**) schematic representation of the steric and electrostatic contributions according to CoMFA using **24** as model compound; (**d**) CoMFA predicted as experimental *p*IC_50_ values.

Owing to the trend observed in supervised multivariate statistics (i.e., single-*Y* OPLS), a three-dimensional quantitative structure-activity relationship (3D-QSAR) study (through a comparative molecular field analysis (CoMFA)) was implemented to correlate structural features and antifungal activity, in order to explain and understand consistently the antifungal activity for test compounds according to steric and electrostatic properties [21]. For this, the best-docked pose of compound **24** was used as template scaffold to define tethers and superimpose the test compounds **1**–**24**. Experimental antifungal data (as logarithmic half-maximal inhibitory concentrations (*p*IC_50_)) and aligned structures were subdivided into a training set (70%) to generate the CoMFA model and a test set (30%) for subsequent external validation [21]. The molecular interaction fields (MIF) were then calculated using probes (steric and electrostatic ones), and, after several steps of data pretreatment, partial least squares (PLS) regression was carried out (using up to five PLS components) in order to build linear relationships between variations in the MIF values as a function of changes in the experimental *p*IC_50_. After that, the best model required three PLS components, suggesting good correlation between MIF values and experimental *p*IC_50_ of test compounds. The resulting regression also afforded the following parameters: correlation coefficient r^2^ = 0.855, a cross-validated leave-one-out (LOO) coefficient q^2^ = 0.779, a cross-validated leave-many-out (LMO) coefficient q^2^ = 0.709 and F-test = 178.489, demonstrating excellent determination coefficient for a statistically predictive, robust model [22]. Observations were randomly organized to perform a *Y*-scrambling protocol [23] using 30 scramblings and 10 runs. The resulting model declined severely (R_scr_^2^ and Q_scr_^2^ < 0.4), and no correlation was then evidenced, indicating the model was not achieved as a result of a chance correlation. Thus, according to this comparative analysis, experimental and CoMFA-based predicted activity for all the compounds, expressed as *p*IC_50_ (Table S4), were used to plot the predicted versus the experimental *p*IC_50_ values for all the test compounds, indicating good correlation for both training and test datasets (Figure 6d). Additionally, this CoMFA model resulted into the electrostatic (35.3%) and steric (29.2%) fields outputs (stdev*coeff), whose translated contour surfaces of the corresponding field contributions, including aligned test compounds, are displayed in Figure 6a–b. 

Regarding the electrostatic field contour map (Figure 6a), the positive effect of positively and negatively-charged regions on antifungal activity can be depicted as blue and red contours, respectively. In the present analysis, electron-deficient substituents were found to be not crucial for the antifungal activity, because any significant blue contour was obtained after the PLS regression. Thus, the activity can be therefore enhanced by the presence of electron-rich groups attached at sulfur in dithiocarbamate moiety, as the electron-withdrawing groups (EWG), and other typical electronegative groups (i.e., heteroatoms). In the case of the contour map of steric field (Figure 6b), unfavorable and favorable zones by steric effects were then symbolized as yellow and green contours, respectively. Thus, the antifungal activity can be enhanced if a bulky steric group is located on the alkyl group (as a large *n*-alkyl substituent) at ester moiety as well as on the EWG, especially in the (3-oxo-1,3-diphenyl)propyl moiety of ODP-DTC-type compounds. In contrast, the antifungal activity is disfavored if a bulky group is placed as a ramification at C2 in the *n*-alkyl group at ester moiety. These results demonstrated that a bulky group with electron-withdrawing nature at sulfur site in dithiocarbamate moiety (such as a chalcone precursor) can improve the antifungal activity. From these facts, a two-dimensional scheme, involving the respective representations of electrostatic and steric effects of test compounds, is exposed in Figure 6c. Accordingly, the resulting model suggested the presence of both bulky and electronegative substituents adequately oriented at the sterically and electrostatically favorable regions, respectively. Both sterically bulky and electron-rich substituents are therefore required together to increase the mycelial growth inhibitory action in test compounds. In this context, compound **24** fitted very well to the above-mentioned structural prerequisites to be a promising antifungal agent. Similar trends were observed from a CoMFA model obtained with close related *N*,*S*-dialkyl dithiocarbamates using other aminoacids (i.e., l-alanine, l-phenylalanine, and l-tyrosine) [9] instead l-tryptophan.

### 2.5. Binding Mode and Residual Interactions

As mentioned above, the behavior of docking scores per enzyme within dataset was found to be different. These differences were found to be depending on the particular enzyme and ligand involved into the docking simulation. Thus, Table S5 exhibited the information of the best-docked phytoalexin analog for each enzyme, which compiles the interacting residues of the respective enzymatic active site and the interacting moieties for the ligands, according to individual two-dimensional residual interaction diagrams (Figure S3). After the analysis of these diagrams to outline some insights into the binding mode of the simulated complexes, several types of interactions were evidenced, involving hydrogen (*H*)-bonds and hydrophobic contacts, common Van der Waals interactions as well as π-π, π-sulfide, π-cation and π-anion interactions. These interactions were taken as the key contacts and an indicative of the importance of the presence of sulfur (as thione) and aromatic rings (indole and EWG) as crucial moieties for interacting with the respective residues of active site and stabilizing the resulting enzyme-ligand complexes. In this regard, compounds **5** and **24** were the best-docked phytoalexin analogs for the corresponding calculation with enzyme E25. The respective complexes (i.e., E25:**5** and E25:**24**) exhibited the lowest Vina scores into the whole dataset (−11.68 ± 0.19 and −13.25 ± 1.02 kcal/mol, respectively), although other good Vina scores were also obtained with these compounds and other enzymes, as previously mentioned (see Figure 3 and Table S2). 

**Figure 7:**
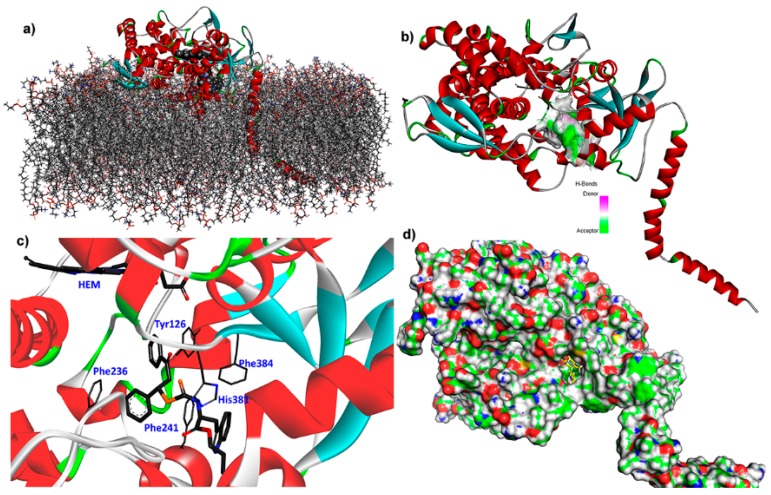
(**a**) *F. cerealis* lanosterol 14α-demethylase (*Fc*LDM), E25, model, embedded in a 266-lipids bilayer of 1-palmitoyl-2-oleoylphosphatidylethanolamine (OPPEA) and docked with compound **24**; (**b**) Interaction surface of E25 and best-docked pose of **24** (contour related to the donor (pink) and acceptor (green) zones with the interface); (**c**) three-dimensional (3D) model of lowest-energy docked pose of **24** within active site of *Fc*LDM (selected residues at active site of *Fc*LDM are marked in gray light sticks; remaining enzyme polycolored cartoon; ligand **24** in gray bold sticks; (**d**) 3D model of solvent-accessible surface area (SASA) of *Fc*LDM with the lowest-energy docked pose of **24** within its active site (ligand **24** in yellow sticks).

E25 is a lanosterol 14α-demethylase (LDM or CYP51), an important enzyme target for several human fungal pathogens. It is a cytochrome P450 enzyme that catalyzes the conversion of lanosterol to 4,4-dimethylcholesta-8(9),14,24-trien-3β-ol (through the C-14α demethylation) [24]. This is a critical step in the sterols biosynthesis, because is the early waypoint in the lanosterol conversion to other important sterols to the cell. Selective inhibition of LDM produces lanosterol accumulation as well as other 14-methyl sterols (often toxic to the fungus), causing fungal growth inhibition [24]. Within the arsenal of fungicides, azoles are reported as selective inhibitors of fungal LDM, such as fluconazole, ketoconazole voriconazole, and itraconazole, frequently used for systemic and topical mycoses [25]. However, the examination of the ergosterol depletion in several, different phytopathogens is still a topic to be explored and extended in order to develop selective inhibitors of this fungal vital pathway. In the case of *Fusarium* spp., the crystal structure of LDM has not been reported yet. Therefore, a homology model was then built using the reported enzyme sequence of the transmembrane LDM from *F. cerealis* (Uniprot entry: I6ZLS0). The resulting homology model of the apoenzyme of *F. cerealis* lanosterol 14α-demethylase (*Fc*LDM) (*Z*-score = 2.191) was embedded in a 266-lipids bilayer of 1-palmitoyl-2-oleoylphosphatidylethanolamine (OPPEA) and SPC water molecules, and chloride and sodium ions, was then optimized through a 5-ns conventional molecular dynamics (MD) simulation. Final optimized model of E25, i.e., *Fc*LDM (Figure 7a), was subsequently used for the above-mentioned molecular docking calculations and further molecular dynamics simulations. Compound **24** exhibited the best Vina score with this enzyme (as above-mentioned) and it exhibited one of the lowest IC_50_ value, indicating its promising behavior as antifungal. Molegro virtual docking (MVD) was also used to simulate this intermolecular interaction as rescoring strategy, and compound **24** exhibited the best score (MVD score = −167.16). Both best-docked poses of **24,** from Autodock/Vina and MVD, exhibited an RMSD below 1, which confirms the good performance of our molecular docking results. 

**Figure 8:**
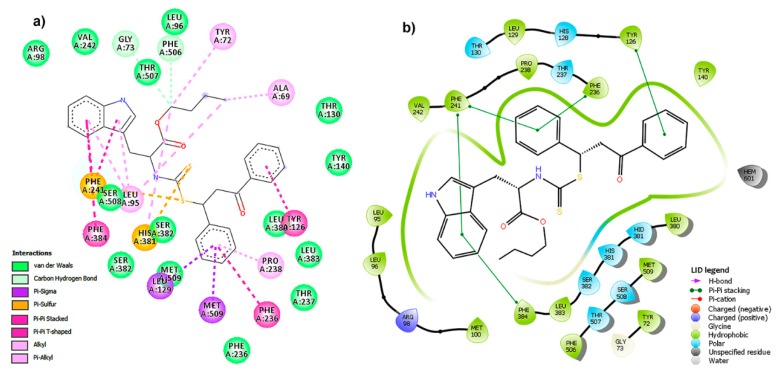
Two-dimensional (2D) plots for *Fc*LDM (E25):**24** complex. (**a**) 2D-residual interaction diagram using Discovery Studio Client (interaction type is differentiated by colored circles (residues) and dashed lines (directed to the ligand moiety) according to the convention); (**b**) ligand interaction diagram (LID) using Maestro 11.5 [light green lines depicts the active site surface, dark green lines indicate the π-π stacking between residue and aromatic moieties of **24**; residues are differentiated by colors according to the interaction type as indicated in LID legend: hydrophobic (green), polar (aquamarine), positive charge (purple)].

This compound fitted adequately within the active site of *Fc*LDM (Figure 7d), located over the membrane for the catalytic action of this transmembrane enzyme (Figure 7a). The *H*-bond interacting surface of the enzyme (Figure 7b) indicated that *Fc*LDM residues can be oriented to have a role as acceptor, but the complex exhibited important non-polar interactions with Tyr126, Phe241, Phe286, His381 and Phe384 residues (Figure 7c). The respective two-dimensional (2D)-residual interaction diagram (Figure 8a) indicated that these key contacts are related to π-sulfur (with His381 and Phe241), π-π stacked (with Tyr126 and Phe236), π-π T-shaped (with Phe384) interaction types. Heme group did not interact with compound **24**, although it is located close to a phenyl group, which implies an advantage in terms of a plausible transformation of the test ligand. In this sense, a successful interaction can be produced, avoiding a posterior catalytic conversion by this HEME group. The ligand interaction plot (Figure 8b) confirms the previous information, indicating Phe241, Phe236, Phe384 and Tyr126 as crucial residues to interact in order to stabilize the ligand-enzyme complex. According to the above analysis, it is possible to conclude that the E25 is a plausible target to be inhibited by **24**, due to its satisfactory in silico interaction profile with this phytoalexin analog. However, this information had to be extended computationally through a molecular dynamics simulation. 

### 2.6. Molecular Dynamics Simulations

Lanosterol 14α-demethylase (LDM) from *F. cerealis* was selected as test enzyme for 90-ns molecular dynamics simulations due to its better molecular docking behavior with the test phytoalexin analogs, specifically compound **24**. The 3D structure of this enzyme was built through homology modeling using Yasara Structure [26], since no crystal data is available, embedded in a 266-lipids OPPEA bilayer. This simulation was implemented as the next step to expand the information about the binding mode of this promising candidate.

**Figure 9:**
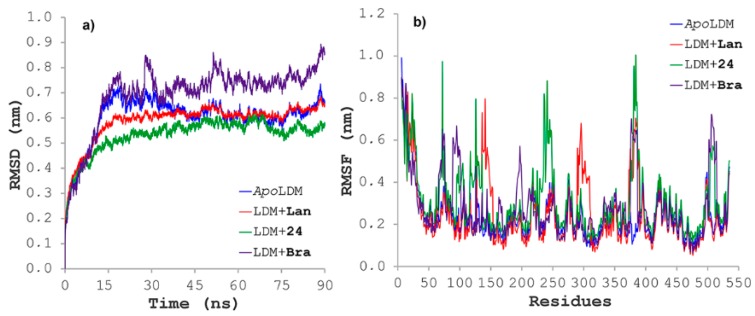
(**a**) Root mean square deviations (RMSD), along the trajectories simulated by molecular dynamics during 90 ns, for the *Fc*LDM (E25) (apoenzyme), and docked separately with **24,** lanosterol (Lan) and brassinin (Bra); (**b**) root mean quadratic fluctuations (RMSF) along the trajectories simulated by molecular dynamics during 90 ns, for the *Fc*LDM (E25) (apoenzyme), and docked separately with **24,** lanosterol (Lan) and brassinin (Bra).

Ligand:protein trajectories for those complexes between **24** and *Fc*LDM were monitored by means of geometric properties over the time. Thus, root mean square deviations (RMSD) were used to evaluate the structural stability of the receptor frame by measuring the time-dependent distance between different positions (in Å) of the set of atoms (Figure 9a). For this, separately 90-ns molecular dynamics simulations for the *Fc*LDM (E25) alone (apoenzyme) and docked distinctly with **24**, lanosterol (Lan) and brassinin (Bra) were recorded for comparing purposes. As result, the apoenzyme exhibited a normal evolution during the initial part of the simulation but revealed a slight perturbation at 20 ns and then a good stabilization over the remaining simulation. Lanosterol avoid such a perturbation and the complex *Fc*LDM:lanosterol reached stability at 15 ns, whereas brassinin displayed the most-perturbed profile. However, the *Fc*LDM:**24** complex achieved early the stability and it is maintained over entire simulation. In addition, the fluctuations of the atomic positions for each residue of the enzyme, the root mean square fluctuation (RMSF) were also considered to scrutinize the flexibility and secondary structure of the *Fc*LDM enzyme under interaction with the above-mentioned test ligands. All examined complexes showed different behavior (Figure 9b), with explicit differences in some regions, fluctuating significantly along the MD simulations trajectory between 0.2 and 1.0 nm. In this regard, test ligands (i.e., brassinin, lanosterol and **24**) exhibited different RMSF profiles, specifically through fluctuations by the particular interacting residues, but a common fluctuating zone was observed (Arg375–Asp385). In the specific case of *Fc*LDM:**24** complex, fluctuations were found to be around the previously identified crucial contacts (i.e., Tyr126, Phe241, Phe286, His381 and Phe384), but the association of **24** did not obviously affect the flexibility of those important fragments to maintain overall system stability (such residues with RMSF > 0.2 nm) in comparison to the *apo*LDM; thus, the inhibition mode may be achieved by stabilizing the system. These results indicated the excellent in silico interaction profile of **24** with the *Fc*LDM enzyme over the time.

### 2.7. Binding Free Energy

The binding free energy for compound **24**, lanosterol and brassinin during interaction of *Fc*LDM for the last 20 ns of MD trajectory was estimated by MM/PBSA approach, using the MD simulations. All three ligands exhibited negative binding energies, but compound **24** showed lower binding energy to that of brassinin (−121.6 ± 7.1 kJ/mol versus −39.7 ± 5.4 kJ/mol, respectively) and lanosterol (−101.3 ± 6.5 kJ/mol), which rationalize the observed best docking performance and the best experimental antifungal activity of **24**. The main contribution to the binding energy was due to vdW energies (> −90 kJ/mol). This fact was then confirmed after binding energy decomposition on the residues of *Fc*LDM:**24** complex, since the most binding energy contributing residues were found to be Phe241 (−9.2 kJ/mol), Phe384 (−4.3 kJ/mol), Phe236 (−3.5 kJ/mol), His381 (−3.2 kJ/mol) and Tyr126 (−2.8 kJ/mol). These results suggested that non-polar electrostatic interactions are the main driving force for molecular recognition of *Fc*LDM by **24**. However, considering the MD simulations and binding affinity calculations, the resulting simulated data should be contemplated carefully, since they were extracted from a single trajectory analysis and the convergence could be hardly obtained for a highly fluctuating system.

## 3. Materials and Methods 

### 3.1. Design and Synthesis of Indole-Containing Phytoalexin Analogs

Twenty-one indole-containing phytoalexin analogs and four L-tryptophan alkyl esters (**1**–**25**) were synthesized through the previously reported protocol [8] (Figure S1) and they were chosen as test compounds (Figure 1). These structures were 3D-sketched and organized according to close-related moieties affording seven substituted groups such as 2-alkyl aminoesters (**1**–**4**), *N*,*N*-dialkylthioureas (**5**–**8**), 2-cyanoethyl *N*-alkyldithiocarbamates (**9**–**12**), 3-methoxy-3-oxopropyl *N*-alkyldithiocarbamate (**13**–**16**), 2-methyl-4-oxopentan-2-yl *N*-alkyldithiocarbamate (**17**–**20**), 2-oxo-1,3-diphenylpropyl *N*-alkyldithiocarbamates (**21**–**24**) and 4-[(1*H*-indol-3-yl)-methylene]-2-sulfanylidene-1,3-thiazolidin-5-one (**25**). Brassinin (**26**) was included into the study and used as reference compound. A previous MMFF geometry minimization for each ligand was carried out in Spartan’14 (Wavefunction, Irvine, CA, USA) without any geometrical restrictions, prior to the docking and dynamics simulations.

### 3.2. Enzymes

Twenty-five fungal enzymes were searched and selected due to their important implications for *Fusarium* spp. in metabolic processes or invasive properties (Table A1). The crystallographic structure of twenty of these enzymes with an inhibitor or substrate was retrieved from the RCBS Protein Data Bank (PDB) (http://www.rcsb.org/). The three-dimensional (3D) structures of the remaining five enzymes were obtained by homology modeling using the standard protocol included into the Yasara Structure package (http://www.yasara.org/) [26]. Water molecules, ligands and other heteroatoms were removed from the protein molecule. Hydrogen atoms were added to the protein. These crystallographic structures were retained without any processing for molecular docking. The co-crystallized inhibitors/substrates were thus employed to define the corresponding active site, the flexible residues within this active site and as a validation criterion of docking calculations (re-docking). PDB-files were then effectively prepared using the AutoDock Vina plugin under PyMOL, and saved as pdbqt-files. The structural information of the employed enzymes is shown in Table A1.

### 3.3. Molecular Docking

Molecular docking was carried out using Autodock/Vina (Molecular Graphics Lab, The Scripps Research Institute, La Jolla, CA, USA) using the AMBER force field with PyMOL 2.3 (Schrödinger LLC, New York, NY, USA) as molecular graphics system. The protein and ligand molecules were prepared as described above. The active site for each enzyme was located into a grid and the flexible residues were selected according to those interacting with co-crystallized inhibitors or substrates at 4.0 Å. All molecular docking calculations were carried out using flexible residues and performed between the optimized ligand into the active site through a cube at the geometrical center of the native ligand present in the evaluated PDB structure, with the dimensions 24 × 24 × 24 Å, covering the ligand binding site with a grid point spacing of 0.375 Å. Each calculation involved ten replicates starting from separated pdb-files of each enzyme and ligand to evaluate convergence. Molecular docking for the co-crystallized inhibitors/substrates was also carried out as a reference to evaluate the docking performance through re-docking. Additionally, the specificity and sensitivity of the docking protocol, 25 compounds of diverse chemical nature, having IC_50_ < 5 μM, against five test enzymes (isocitrate lyase, aspartate kinase, sereine esterase, lanosterol 14α-demethylase and trichothecene acetyltransferase) were compiled from the literature and available databases such as ChEMBL [27] and pubchem [28]. Forty-five decoys per each active compound were compiled from the directory of useful decoys (DUD-E) [29] and CheMBL databases. Thus, decoys and bioactives were processed using the docking process. The resulting data was then assessed through a receiver operating characteristic (ROC) and scores enrichment using the screening explorer webserver [30]. Once the docking parameters were validated, the docking simulations of test indole-containing phytoalexin analogs were performed. The resulting binding modes were firstly analyzed in PyMOL 2.3 and the docking poses were ranked according to their docking Vina scores (as affinities in kcal/mol). Molegro Virtual Docker 6.0 (CLC Bio Company, Aarhus, Denmark) was also used to evaluate secondly the best-poses of best-docked compounds as a rescoring strategy. Both the ranked list of docked ligands and their corresponding binding poses were exported as a CSV file for further analysis. Moreover, two-dimensional (2D) residual interactions diagrams and 3D figures of these enzymes were obtained using Discovery Studio Client 16.1 (Biovia, San Diego, CA, USA) and Maestro Elements 8.3 (Schrödinger, Cambridge, MA, USA) from the Vina outputs (pdbqt-files) for compounds that exhibited highest affinity. All calculations were performed in a Dual Intel Xeon^®^ processor CPU @ 2.6 GHz of Intel system origin, with 64 GB DDR3 RAM on an Ubuntu 12.04 server.

### 3.4. Statistical Analysis

Descriptive and inferential statistics tests were carried out such as principal component analysis, significance tests and partial least-square (PLS) regression. Orthogonal partial least squares-discriminant analysis (OPLS-DA) and single-*Y* OPLS were carried out in SIMCA 14.0 software (Umetrics, Umeå, Sweden) using the docking affinity values and the reported IC_50_ values in order to observe feasible relationships.

### 3.5. Molecular Dynamics Simulations of F. Cerealis Lanosterol 14α-demethylase (FcLDM)

The resulting homology model of the apoenzyme of *F. cerealis* lanosterol 14α-demethylase (*Fc*LDM) were embedded in a 1-palmitoyl-2-oleoylphosphatidylethanolamine (OPPEA) bilayer of 266 lipids covering an area of 125 × 125 Å^2^, using a cell box with size of 125 × 125 × 200 Å^3^ filled with SPC water molecules for solvation. The net protein charge was neutralized by 60 chloride and 54 sodium ions. This membrane-*Fc*LDM model was built using the standard protocol included into the Yasara Structure package [26]. The most plausible model was identified through steepest descent method-based energy minimization until 100 kJ/(mol·nm) energy convergence, followed by a 250-ps restrained equilibration simulation and a 5-ns conventional molecular dynamics (MD) simulation. The same protocol was carried out on the simulation systems of *Fc*LDM+lanosterol, *Fc*LDM+brassinin and *Fc*LDM + **24** (obtained after molecular docking), which were individually subjected to an additional 90-ns conventional MD simulation for exploring the performance of these ligands within the active site of the enzyme. These MD simulations were run in Gromacs 5.0.5 (open source, http://www.gromacs.org) on an Ubuntu 12.04 server, using NPT and periodic boundary conditions, as previously reported [31]. Hence, docked ligands were prepared by adding hydrogen atoms in UCSF Chimera (UCSF, CA, USA) and the resulting pdb-file was uploaded to the atb server (http://compbio.biosci.uq.edu.au/atb/) to add the respective Gromo53a6 force field and get the itp-type topology file. Membrane-*Fc*LDM topologies were obtained in Gromacs using the Gromos53a6 force fields, owing to the presence of Heme group. The SPC water model was then implemented for solvation, in a triclinic box using a 1.0-nm margin distance. 0.10 M NaCl was added to the simulation systems and water molecules were randomly replaced until neutrality. An energy minimization through 2000-steps steepest descent method was then used. NVT equilibration at 310 K during 500 ps, followed by NPT equilibration during 1000 ps using the Parrinello-Rahman method at 1 bar as reference were done on the systems using position restraints. Finally, solute position restraints were released and a production run for 90 ns was performed. Temperature and pressure were kept constant at 310 K and 1 bar, respectively. Coordinates were recorded in a 1 fs time step. Electrostatic forces were calculated using the particle-mesh Ewald (PME) method. Periodic boundary conditions were used in all simulations and covalent bond lengths were constrained by the LINCS algorithm.

### 3.6. Binding Free Energy Analyses

Binding free energy was calculated using the g_mmpbsa tool [32](open source drug discovery consortium (OSDD), New Dehli, India). This tool calculated components of the free energy of the protein–substrate binding (ΔG_Bind_), using the molecular mechanic/poisson-boltzmann surface area (MM/PBSA) method [33,34]. In this method, ΔG_bind_ calculation between a protein and a ligand is carried out by ∆G_bind_ = ∆H − T∆S ≈ ∆E_MM_ + ∆G_sol_ − T∆S, ∆E_MM_ = ∆E_internal_ + ∆E_electrostatic_ − ∆E_vdW_, ∆Gsolv = ∆G^elec^_solv_ + DG^vdW^_solv_. Here, the total gas phase energy on the binding of MM energy is ΔE_MM_, the free energy of solvation is ΔG_solv_ and the entropy contribution is TΔS. Poisson-Boltzmann model was used to compute the electrostatic solvation energy in a continuum solvent. The derivation of non-polar solvation energy term was computed as solvent-accessible surface area (SASA). ΔE_MM_ were calculated using the Lennard-Jones and Coulomb potential [33,34]. ΔG_Bind_ was used to analyze the binding associations between *Fc*LDM and selected ligands (i.e., lanosterol, **24** and brassinin) by decomposing the total binding free energy into each residue. The binding energy calculations of the selected ligands were performed for 100 snapshots taken at an interval of 200 ps during the last sTable 20-ns MD simulations.

### 3.7. Antifungal Assay

Compounds **1**–**25** were evaluated for their antifungal activity against *F. oxysporum* following the previously reported amended-medium protocol to assess the in-vitro mycelial growth inhibition [8]. The activity was expressed as half-maximal inhibitory concentrations, IC_50_ (in mM), obtained after non-linear regression using the program GraphPad Prism version 5.00 (GraphPad Software, San Diego, CA, USA).

### 3.8. Comparative Molecular Field Analysis (CoMFA)

Best-docked poses of test ligands were merged and aligned by means of particular tethers placed on indole, sulfur and aromatic rings moieties, using the molecular overlay tool included in the software Discovery Studio Client 16.1 (Biovia, San Diego, CA, USA). The best-scored compound (i.e., 24) was selected as template to select tethers. The aligned molecules set (in a sdf-file) was randomly divided into two subsets (training and test sets, corresponding to 70 and 30%, respectively), and CoMFA analysis was then performed using Open3DQSAR, using the standard protocol [35]. The experimental antifungal activity evaluated as *F. oxysporum* mycelial growth inhibition (expressed as IC_50_ in M) were was converted into negative logarithmic form (*p*IC_50_) and then used as independent variable. The models were validated by leave-one-out (LOO) and leave-many-out (LMO) methods. The quality of the models after validation was evaluated, predicting the independent variable for the test set. 

## 4. Conclusions

Twenty-one indole-containing phytoalexin analogs and four L-tryptophan alkyl esters (**1**–**25**) were docked within the active site of a set of 25 enzymes of *Fusarium* spp. through Autodock/Vina. NNDATU, ODP-TDC and IST-type analogs exhibited the best docking scores and interaction profile with several enzymes, especially nitroalkane oxidases (E17 and E18) and FMN-dependent dehydrogenase (E24) and 14-lanosterol demethylase (E25), respectively, according to the distribution and correlation analyses as well as discriminant analysis by OPLS. Computational results were also integrated to experimental in vitro (IC_50_) mycelial growth inhibition against *F. oxysporum*, exhibiting good correlations between docking scores and antifungal data (by single-Y OPLS) as well as among structures and antifungal data (on the basis of a robust CoMFA model). The CoMFA-derived PLS-regression indicated that sterically bulky and electron-rich substituents are jointly required to increase the mycelial growth inhibitory action in test compounds, indicating ODP-TD-type compounds can be considered as candidates for phytoalexin bioisosteres. E25 (a homology model of a *F. cerealis* 14-lanosterol demethylase) was taken as study model owing to the well-behaved interaction with compound **24** (*tert*-butyl (((3-oxo-1,3-diphenylpropyl)thio)carbonothioyl)-L-tryptophanate). The binding mode was examined, and some residues were found to be important to stabilize the *Fc*LDM:**24** complex, which was extended over the time by 90-ns molecular dynamics simulations. In this regard, the non-polar electrostatic interactions can be considered as the main driving force for the molecular recognition of *Fc*LDM by **24**. From all of the obtained results, compound **24** can be considered as a putative inhibitor of this enzyme, rationalizing the good experimental antifungal activity through in vitro mycelial growth inhibition (IC_50_ = 0.44 mM). Such a mode of action will be further explored by in vitro enzymatic inhibition over purified enzyme, which is currently ongoing. Thus, after data analyses, some important hits, putative enzyme targets and structural requirements were established, indicating the potential of indole-containing phytoalexin-based bioisosteres as an alternative for the control of *Fusarium* spp.-like phytopathogens. Hence, this information will be preserved during our further studies focused on the development of antifungals based on phytoalexin analogs.

## Figures and Tables

**Table 1:** Values of Pearson correlation for subsets of test compounds.

CC *^a^*	C1	C2	C3	C4	C5	C6	C7
**C1**	1.00						
**C2**	0.699	1.00					
**C3**	0.872	0.832	1.00				
**C4**	0.926	0.731	0.901	1.00			
**C5**	0.921	0.747	0.886	0.985	1.00		
**C6**	0.647	0.626	0.627	0.678	0.724	1.00	
**C7**	0.928	0.704	0.886	0.912	0.888	0.636	1.00
**C8**	0.728	0.444	0.670	0.693	0.671	0.446	0.694

*^a^* Compound classes: **C1**: alkyl 2-aminoesters (AAE); **C2**: *N*,*N*-dialkylthioureas (NNDATU); **C3:** 2-cyanoethyl *N*-alkyldithiocarbamates (CE-DTC); **C4**: 3-methoxy-3-oxopropyl *N*-alkyldithiocarbamate (MOPr-DTC), **C5:** 2-methyl-4-oxopentan-2-yl *N*-alkyldithiocarbamate (MOPe-DTC); **C6:** 2-oxo-1,3-diphenylpropyl *N*-alkyldithiocarbamates (ODP-DTC), **C7:** 4-[(1*H*-indol-3-yl)-methylene]-2-sulfanylidene-1,3-thiazolidin-5-one (IST); **C8:** brassinin.

## Supplementary Materials

The following are available online at https://www.mdpi.com/1420-3049/25/1/45/s1, Table S1: IUPAC names of test phytoalexin analogues. Table S2: Calculated Vina scores for compounds 1–26 docked with enzymes E1–E25. Table S3: Values for the Pearson correlation of the enzymes set. Table S4: Antifungal activity against *Fusarium oxysporum* through amended-medium assay. Table S5: Recorded interactions according to the best-docked pose of test phytoalexin analogs. Figure S1: Reaction route for obtaining the test indole-containing phytoalexin analogs. Figure S2: Principal component analysis (PCA)-derived score plot. Unsupervised multivariate analysis from affinity dataset. Figure S3: 2D-residual interactions for the test indole-containing phytoalexin analogs.

## Appendix A

**Table A1:** Data of test enzymes.

ID	Enzyme (Type)	PDB Code	Source
E1	endoglucanase I (glycosyl hydrolase) [NAG cavity] ^a^	1OVW	*F. oxysporum*
E2	endoglucanase I (glycosyl hydrolase) [native binding site cavity] ^a^	1OVW	*F. oxysporum*
E3	endoglucanase I (glycosyl hydrolase)	4OVW	*F. oxysporum*
E4	endoglucanase I (cellobiohydrolase)	3OVW	*F. oxysporum*
E5	acetyltransferase (transferase)	2ZBA	*F. sporotrichioides*
E6	thrichodiene synthase (synthase)	2PS8	*F. sporotrichioides*
E7	endoglucanase I (cellobiohydrolase)	2OVW	*F. oxysporum*
E8	trichodiene synthase (synthase)	1YYQ	*F. sporotrichioides*
E9	serine esterase (cutinase)	1XZM	*F. solani* subsp. pisi
E10	serine esterase (hydrolase-cutinase)	1XZL	*F. solani* subsp. pisi
E11	serine esterase (cutinase)	1OXM	*F. solani* subsp. pisi
E12	isocitrate lyase (lyase)	5E9G	*F. sporotrichioides*
E13	trichothecene 15-O-acetyltransferase (transferase)	3FP0	*F. sporotrichioides*
E14	isocitrate lyase (lyase)	5E9H	*F. graminearum*
E15	trichodiene synthase (synthase)	1JFG	*F. oxysporum*
E16	trichothecene 3-O-acetyltransferase (transferase)	3B30	*F. graminearum*
E17	nitroalkane oxidase (NAO) (oxidoreductase)	2REH	*F. oxysporum*
E18	nitroalkane oxidase (NAO) (oxidoreductase)	2C0U	*F. oxysporum*
E19	flavoenzyme nitroalkane oxidase (oxidoreductase)	3D9E	*F. oxysporum*
E20	nitric oxide reductase cytochrome (oxidoreductase)	1ULW	*F. oxysporum*
E21	NAD(P)-dependent dehydrogenase (dehydrogenase)	1U3T ^b^	*F. vascular (A0A0D2YG03 ^c^)*
E22	nitroreductase [NADPH] (oxidoreductase)	2BII ^b^	*F. vascular wilt (P39863 ^c^)*
E23	aspartate kinase (kinase)	2CDQ ^b^	*F. verticilloides (W7MS01 ^c^)*
E24	FMN-dependent dehydrogenase (oxidoreductase)	1GOX ^b^	*F. verticilloides (W7NCP1 ^c^)*
E25	14-lanosterol demethylase (CYP51) (oxidoreductase)	4LXJ ^b^	*F. cerealis (I6ZLS0 ^c^)*

^a^ Two different cavities (binding pockets) evaluated of the same enzyme structure; ^b^ Enzymes taken as template (after sequence alignment through homology modeling) from respective Uniprot sequences of *Fusarium* enzymes using Yasara Structure package [9]; ^c^ Uniprot entries of enzyme sequences.
