# Supplementary material for: Indole-Containing Phytoalexin-Based Bioisosteres as Antifungals: In Vitro and In Silico Evaluation against Fusarium oxysporum
Source: Molecules. 2019 Dec 21;25(1):45. doi: 10.3390/molecules25010045 (PMC6982726; doi:10.3390/molecules25010045)
Supplement: Supplementary file 1 [file molecules-25-00045-s001.pdf]

Supplementary Information

Indole-Containing Phytoalexin-Based Bioisosteres as Antifungals: In Vitro and In Silico Evaluation against *Fusarium oxysporum*

Andrea Angarita-Rodríguez[1*], Diego Quiroga[1], Ericsson Coy-Barrera[1*]

[1]Bioorganic Chemistry Laboratory, Facultad de Ciencias Básicas y Aplicadas, Universidad Militar Nueva Granada, Campus Nueva Granada, Cajicá, 250247, Colombia

Content

		Page
**Table S1.**	IUPAC names of test phytoalexin analogues	2
**Table S2.**	Calculated Vina scores for compounds **1-26** docked with enzymes E1-E25.	3
**Table S3.**	Values for the Pearson correlation of the enzymes set.	6
**Table S4.**	Antifungal activity against *Fusarium oxysporum* through amended-medium assay	7
**Table S5.**	Recorded interactions according to the best-docked pose of test phytoalexin analogs	8
**Figure S1.**	Reaction route for obtaining the test indole-containing phytoalexin analogues	9
**Figure S2.**	PCA-derived score plot. Unsupervised multivariate analysis from affinity dataset	10
**Figure S3.**	2D-residual interactions for the test indole-containing phytoalexin analogues.	11

Table S1. IUPAC names of test phytoalexin analogues

Compound number	IUPAC name
**1**	methyl *L*-tryptophanate
**2**	ethyl *L*-tryptophanate
**3**	isopropyl *L*-tryptophanate
**4**	*tert*-butyl *L*-tryptophanate
**5**	dimethyl 2,2'-(thiocarbonylbis(azanediyl))(2*S*,2'*S*)-bis(3-(1*H*-indol-3-yl)propanoate)
**6**	diethyl 2,2'-(thiocarbonylbis(azanediyl))(2*S*,2'*S*)-bis(3-(1*H*-indol-3-yl)propanoate)
**7**	diisopropyl 2,2'-(thiocarbonylbis(azanediyl))(2*S*,2'*S*)-bis(3-(1*H*-indol-3-yl)propanoate)
**8**	di-*tert*-butyl 2,2'-(thiocarbonylbis(azanediyl))(2*S*,2'*S*)-bis(3-(1*H*-indol-3-yl)propanoate)
**9**	methyl (((2-cyanoethyl)thio)carbonothioyl)-*L*-tryptophanate
**10**	ethyl (((2-cyanoethyl)thio)carbonothioyl)-*L*-tryptophanate
**11**	isopropyl (((2-cyanoethyl)thio)carbonothioyl)-*L*-tryptophanate
**12**	*tert*-butyl (((2-cyanoethyl)thio)carbonothioyl)-*L*-tryptophanate
**13**	methyl (((3-methoxy-3-oxopropyl)thio)carbonothioyl)-*L*-tryptophanate
**14**	ethyl (((3-methoxy-3-oxopropyl)thio)carbonothioyl)-*L*-tryptophanate
**15**	isopropyl (((3-methoxy-3-oxopropyl)thio)carbonothioyl)-*L*-tryptophanate
**16**	*tert*-butyl (((3-methoxy-3-oxopropyl)thio)carbonothioyl)-*L*-tryptophanate
**17**	methyl (((2-methyl-4-oxopentan-2-yl)thio)carbonothioyl)-*L*-tryptophanate
**18**	ethyl (((2-methyl-4-oxopentan-2-yl)thio)carbonothioyl)-*L*-tryptophanate
**19**	isopropyl (((2-methyl-4-oxopentan-2-yl)thio)carbonothioyl)-*L*-tryptophanate
**20**	*tert*-butyl (((2-methyl-4-oxopentan-2-yl)thio)carbonothioyl)-*L*-tryptophanate
**21**	methyl (((3-oxo-1,3-diphenylpropyl)thio)carbonothioyl)-*L*-tryptophanate
**22**	ethyl (((3-oxo-1,3-diphenylpropyl)thio)carbonothioyl)-*L*-tryptophanate
**23**	isopropyl (((3-oxo-1,3-diphenylpropyl)thio)carbonothioyl)-*L*-tryptophanate
**24**	tert-butyl (((3-oxo-1,3-diphenylpropyl)thio)carbonothioyl)-*L*-tryptophanate
**25**	(*Z*)-4-((1*H*-indol-3-yl)methylene)-2-thioxothiazolidin-5-one
**26**	methyl ((1*H*-indol-3-yl)methyl)carbamodithioate (brassinin)

Table S2. Calculated Vina scores (kcal/mol) for compounds **1-26** docked with enzymes E1-E25.

**Compound**	**Type***	**E1**	**E2**	**E3**	**E4**	**E5**	**E6**	**E7**	**E8**
**1**	AAE	-5.14 ±0.04	-7.59 ±0.17	-6.94 ±0.06	-4.92 ±0.04	-6.61 ±0.02	-6.19 ±0.15	-8.51 ±0.13	-6.35 ±0.25
**2**	AAE	-5.15 ±0.03	-7.44 ±0.16	-6.98 ±0.09	-4.92 ±0.05	-6.64 ±0.03	-6.16 ±0.16	-7.07 ±0.13	-6.26 ±0.08
**3**	AAE	-5.23 ±0.04	-7.93 ±0.16	-7.81 ±0.48	-5.04 ±0.08	-7.10 ±0.03	-6.28 ±0.26	-7.23 ±0.07	-6.59 ±0.11
**4**	AAE	-5.29 ±0.05	-8.25 ±0.04	-7.48 ±0.06	-4.79 ±0.07	-7.17 ±0.06	-6.56 ±0.19	-7.39 ±0.05	-6.54 ±0.16
**5**	NNDATU	-4.65 ±0.38	-9.56 ±0.31	-9.24 ±0.37	-5.04 ±0.07	-8.27 ±0.32	-8.09 ±0.30	**-8.61 ±0.10**	-8.21 ±0.26
**6**	NNDATU	-4.41 ±0.17	-9.44 ±0.18	-9.31 ±0.28	-4.86 ±0.12	-8.00 ±0.29	-7.83 ±0.34	-8.36 ±0.11	-8.17 ±0.24
**7**	NNDATU	-4.69 ±0.40	-9.32 ±0.99	-9.47 ±0.45	-4.94 ±0.11	-8.14 ±0.11	-8.22 ±0.32	**-8.68 ±0.20**	-8.01 ±0.29
**8**	NNDATU	-5.40 ±0.56	-9.40 ±0.14	-9.47 ±0.28	-5.00 ±0.09	-8.08 ±0.09	-8.14 ±0.20	-8.48 ±0.06	-8.44 ±0.27
**9**	CE-DTC	-4.91 ±0.18	-7.64 ±0.13	-7.5 ±0.21	-4.7 ±0.17	-7.56 ±0.26	-6.71 ±0.18	-7.02 ±0.22	-6.82 ±0.14
**10**	CE-DTC	-4.91 ±0.18	-7.32 ±0.20	-7.58 ±0.20	-4.65 ±0.19	-7.47 ±0.18	-8.13 ±0.04	-6.76 ±0.09	-5.95 ±0.12
**11**	CE-DTC	-4.94 ±0.13	-7.99 ±0.32	-7.60 ±0.20	-4.98 ±0.21	-7.75 ±0.28	-7.37 ±0.38	-7.33 ±0.22	-6.98 ±0.43
**12**	CE-DTC	-4.97 ±0.13	-7.73 ±0.33	-7.57 ±0.39	-4.79 ±0.09	-7.36 ±0.16	-8.08 ±0.22	-7.20 ±0.09	-5.95 ±0.38
**13**	MOPr-DTC	-5.13 ±0.17	-7.73 ±0.24	-7.52 ±0.21	-4.93 ±0.16	-7.61 ±0.14	-6.51 ±0.10	-6.86 ±0.19	-6.82 ±0.17
**14**	MOPr-DTC	-5.11 ±0.16	-7.39 ±0.24	-7.57 ±0.26	-4.83 ±0.19	-7.40 ±0.18	-6.72 ±0.14	-6.98 ±0.21	-6.83 ±0.14
**15**	MOPr-DTC	-5.19 ±0.20	-7.62 ±0.16	-7.46 ±0.33	-4.91 ±0.23	-7.54 ±0.2	-6.60 ±0.41	-6.98 ±0.14	-6.82 ±0.23
**16**	MOPr-DTC	-5.16 ±0.24	-7.80 ±0.11	-7.58 ±0.11	-5.00 ±0.06	-7.51 ±0.11	-7.16 ±0.12	-7.08 ±0.18	-7.39 ±0.24
**17**	MOPe-DTC	-5.19 ±0.14	-7.48 ±0.27	-7.53 ±0.28	-4.96 ±0.18	-7.50 ±0.13	-6.87 ±0.24	-7.03 ±0.17	-6.93 ±0.30
**18**	MOPe-DTC	-4.97 ±0.21	-7.84 ±0.06	-7.46 ±0.24	-4.69 ±0.13	-7.72 ±0.13	-6.97 ±0.22	-7.20 ±0.12	-7.14 ±0.16
**19**	MOPe-DTC	-4.45 ±0.11	-8.06 ±0.07	-7.70 ±0.21	-4.79 ±0.19	-7.76 ±0.13	-7.21 ±0.16	-7.33 ±0.18	-7.22 ±0.22
**20**	MOPe-DTC	-4.81 ±1.69	-8.01 ±0.14	-7.79 ±0.16	-4.79 ±0.16	-7.76 ±0.12	-7.34 ±0.19	-7.40 ±0.19	-7.53 ±0.10
**21**	ODP-DTC	-5.35 ±0.11	**-10.10 ±0.25**	-9.51 ±0.5	-5.50 ±0.08	**-9.02 ±0.25**	**-8.95 ±0.09**	-8.50 ±0.06	-8.80 ±0.16
**22**	ODP-DTC	-5.34 ±0.11	**-10.14 ±0.31**	**-9.66 ±0.52**	-5.41 ±0.04	-8.65 ±0.07	-8.83 ±0.13	-8.44 ±0.08	-8.71 ±0.21
**23**	ODP-DTC	**-5.45 ±0.08**	-9.93 ±0.27	-9.49 ±0.42	-5.54 ±0.03	-8.20 ±0.16	**-9.00 ±0.22**	-8.48 ±0.09	**-8.88 ±0.40**
**24**	ODP-DTC	-5.40 ±0.12	-10.04 ±0.19	**-9.86 ±0.52**	**-5.65 ±0.11**	-8.19 ±0.16	-8.78 ±0.29	-8.51 ±0.13	**-8.96 ±0.24**
**25**	IST	**-5.73 ±0.01**	-8.50 ±0.06	-8.35 ±0.24	**-5.61 ±0.02**	-7.49 ±0.04	-8.12 ±0.05	-8.12 ±0.05	-6.28 ±0.06
**26**	Brassinin	-4.76 ±0.08	-8.99 ±0.46	-6.78 ±0.46	-4.61 ±0.25	**-8.70 ±0.26**	-4.97 ±0.14	-5.87 ±0.30	-4.57 ±0.38

*alkyl 2-aminoesters (**AAE**), *N*,*N*-dialkylthioureas (**NNDATU**), 2-cyanoethyl *N*-alkyldithiocarbamates (**CE-DTC**), 3-methoxy-3-oxopropyl *N*-alkyldithiocarbamate (**MOPr-DTC**), 2-methyl-4-oxopentan-3-yl *N*-alkyldithiocarbamate (**MOPe-DTC**), 2-oxo-1,3-diphenylpropyl *N*-alkyldithiocarbamates (**ODP-DTC**), 4-[(1*H*-indol-3-yl)-methylene]-2-sulfanylidene-1,3-thiazolidin-5-one (**IST**).

Table S2. Calculated Vina scores (kcal/mol) for compounds **1-26** docked with enzymes E1-E25 (*cont.*).

Compound	Type*	E9	E10	E11	E12	E13	E14	E15	E16
**1**	AAE	-5.37±0.18	-5.79±0.06	-6.44±0.06	-6.29±0.25	-5.49±0.26	**-4.68±0.06**	-6.70±0.16	-6.29±0.25
**2**	AAE	-5.43±0.20	-5.78±0.03	-6.5±0.11	-6.46±0.28	-5.28±0.21	**-4.60±0.06**	-6.66±0.18	-6.46±0.28
**3**	AAE	-5.43±0.05	-6.35±0.16	-7.10±0.07	-6.69±0.14	-5.26±0.12	-4.49±0.07	-6.92±0.06	-6.61±0.13
**4**	AAE	-5.51±0.04	-6.71±0.36	-7.07±0.37	-7.00±0.14	-5.49±0.19	-4.42±0.16	-7.01±0.11	-6.98±0.16
**5**	NNDATU	-5.32±0.14	-5.97±0.56	-7.99±0.15	-6.89±0.23	-5.59±0.63	-3.72±2.23	**-8.60±0.47**	-6.93±0.21
**6**	NNDATU	-5.30±0.22	-6.45±0.32	-7.80±0.22	**-7.27±0.49**	-5.87±0.72	-0.06±1.67	-8.36±0.47	-7.27±0.50
**7**	NNDATU	-5.15±0.10	-5.69±0.22	-8.14±0.15	-6.90±0.19	-5.91±0.65	-0.06±1.67	-8.23±0.66	-6.86±0.20
**8**	NNDATU	-5.66±0.32	-6.07±0.56	-8.13±0.50	-7.13±0.57	-6.03±0.63	-0.75±1.86	-7.80±0.39	-7.23±0.40
**9**	CE-DTC	-5.43±0.03	-6.05±0.12	-6.76±0.09	-6.27±0.28	-5.19±0.16	-4.11±0.30	-7.00±0.21	-6.26±0.29
**10**	CE-DTC	-5.49±0.10	-5.91±0.18	-6.82±0.23	-5.99±0.23	-5.23±0.09	-3.68±0.23	-7.03±0.24	-5.97±0.19
**11**	CE-DTC	-5.17±0.22	-5.92±0.23	-7.44±0.14	-6.55±0.18	-5.39±0.19	-2.93±0.74	-7.47±0.20	-6.52±0.17
**12**	CE-DTC	-5.21±0.23	-5.84±0.12	-7.24±0.34	-6.36±0.25	-5.29±0.16	-2.64±0.82	-7.28±0.28	-6.31±0.18
**13**	MOPr-DTC	-5.19±0.15	-5.76±0.22	-6.77±0.19	-6.92±0.27	-5.14±0.09	-3.32±2.11	-7.28±0.17	-6.95±0.25
**14**	MOPr-DTC	-5.12±0.26	-5.99±0.13	-6.85±0.15	-6.60±0.37	-5.05±0.14	-3.60±0.50	-7.31±0.30	-6.58±0.35
**15**	MOPr-DTC	-5.24±0.07	-5.85±0.25	-6.88±0.25	-6.46±0.42	-5.16±0.15	-3.02±0.68	-7.40±0.20	-6.49±0.43
**16**	MOPr-DTC	-5.42±0.15	-5.90±0.31	-7.30±0.22	-6.66±0.18	-5.26±0.11	-2.15±1.84	-7.39±0.23	-6.65±0.19
**17**	MOPe-DTC	-5.26±0.15	-5.76±0.26	-7.16±0.15	-6.50±0.27	-5.24±0.13	-2.23±1.84	-7.37±0.35	-6.54±0.22
**18**	MOPe-DTC	-5.24±0.17	-5.53±0.25	-7.32±0.12	-6.25±0.40	-5.18±0.21	-3.66±0.50	-7.47±0.23	-6.18±0.38
**19**	MOPe-DTC	-5.10±0.21	-6.20±0.34	-7.47±0.09	-6.47±0.34	-5.50±0.15	-3.03±0.41	-7.51±0.33	-6.52±0.20
**20**	MOPe-DTC	-4.85±0.43	-6.38±0.49	-7.58±0.38	-6.61±0.38	-5.58±0.25	-0.34±2.60	-7.90±0.16	-6.44±0.34
**21**	ODP-DTC	**-6.00±0.10**	**-7.33±0.45**	**-8.25±0.23**	-7.00±0.18	-6.07±0.31	0.81±1.53	**-8.63±0.46**	-7.06±0.23
**22**	ODP-DTC	-5.67±0.10	**-7.31±0.75**	-8.15±0.13	-6.97±0.32	-6.18±0.33	0.55±1.06	-8.38±0.44	-6.95±0.32
**23**	ODP-DTC	-5.99±0.07	-6.11±0.25	**-8.20±0.13**	-7.18±0.11	**-6.23±0.27**	1.18±1.22	-8.30±0.50	-7.18±0.11
**24**	ODP-DTC	**-6.03±0.12**	-6.41±0.20	-8.08±0.31	**-7.33±0.16**	**-6.36±0.31**	0.79±2.27	-8.63±0.61	**-7.28±0.10**
**25**	IST	-5.66±0.01	-6.28±0.06	-7.28±0.02	**-7.50±0.20**	-6.00±0.13	-5.23±0.1	-7.94±0.10	**-7.53±0.21**
**26**	Brassinin	-4.73±0.16	-4.46±0.21	-4.52±0.15	-4.34±0.12	-4.80±0.23	-4.33±0.14	-6.14±0.13	-5.77±0.26

*alkyl 2-aminoesters (**AAE**), *N*,*N*-dialkylthioureas (**NNDATU**), 2-cyanoethyl *N*-alkyldithiocarbamates (**CE-DTC**), 3-methoxy-3-oxopropyl *N*-alkyldithiocarbamate (**MOPr-DTC**), 2-methyl-4-oxopentan-3-yl *N*-alkyldithiocarbamate (**MOPe-DTC**), 2-oxo-1,3-diphenylpropyl *N*-alkyldithiocarbamates (**ODP-DTC**), 4-[(1*H*-indol-3-yl)-methylene]-2-sulfanylidene-1,3-thiazolidin-5-one (**IST**).

Table S2. Calculated Vina scores (kcal/mol) for compounds **1-26** docked with enzymes E1-E25 (*cont.*).

Compound	Type*	E17	E18	E19	E20	E21	E22	E23	E24	E25
**1**	AAE	-7.05±0.05	-7.15±0.04	-6.90±0.51	**-8.46±0.02**	-6.72±0.53	-7.49±0.04	-6.46±0.03	-7.26±0.01	-7.20±0.20
**2**	AAE	-7.19±0.04	-7.57±0.04	-6.90±0.51	-6.08±0.02	-7.15±0.40	-7.14±0.12	-6.28±0.03	-7.43±0.04	-7.35±0.13
**3**	AAE	-7.47±0.05	-7.69±0.12	-7.53±0.14	-6.54±0.03	-7.31±0.35	-7.28±0.17	-6.48±0.02	-7.47±0.04	-7.58±0.22
**4**	AAE	-7.76±0.03	-7.88±0.05	-8.06±0.15	-6.57±0.04	-7.08±0.28	-7.36±0.18	-6.78±0.08	-7.74±0.05	-7.98±0.31
**5**	NNDATU	-10.43±0.07	**-10.85±0.10**	-9.98±0.13	-6.69±0.22	-3.45±1.50	-7.05±0.12	-6.79±0.33	-9.44±0.01	**-11.68±1.02**
**6**	NNDATU	-10.45±0.05	**-10.82±0.19**	-9.29±0.10	-6.31±0.45	-4.56±0.10	-7.33±0.15	-7.11±0.48	-9.42±0.40	-10.55±0.12
**7**	NNDATU	**-10.70±0.05**	-10.53±0.04	-9.06±0.10	-6.64±0.37	-4.69±0.11	-8.39±0.25	**-7.55±0.34**	**-10.03±0.09**	-10.8±0.19
**8**	NNDATU	-9.54±3.35	-10.54±0.08	-8.48±0.10	-6.58±0.28	-4.63±0.09	-8.44±0.15	-7.21±0.18	**-9.78±0.03**	-10.66±0.23
**9**	CE-DTC	-8.78±0.14	-9.11±0.07	-8.21±0.08	-6.46±0.15	-5.12±0.13	**-8.56±0.19**	**-7.33±0.21**	-8.09±0.07	-8.01±0.37
**10**	CE-DTC	-8.21±0.16	-9.08±0.06	-8.11±0.14	-6.14±0.25	-7.53±0.42	-7.46±0.03	-6.11±0.22	-8.10±0.07	-7.99±0.29
**11**	CE-DTC	-9.09±0.07	-9.47±0.14	-8.15±0.18	-6.71±0.31	-7.77±0.33	-7.43±0.01	-6.12±0.15	-8.67±0.02	-8.58±0.21
**12**	CE-DTC	-8.74±0.07	-8.94±0.06	-8.26±0.16	-6.26±0.18	-7.71±0.32	-7.73±0.04	-6.24±0.10	-7.99±0.07	-8.34±0.20
**13**	MOPr-DTC	-8.27±0.13	-9.12±0.05	-8.21±0.15	-6.59±0.34	-7.67±0.42	-7.65±0.04	-6.32±0.15	-8.45±0.04	-7.99±0.22
**14**	MOPr-DTC	-8.55±0.11	-9.17±0.07	-8.36±0.13	-6.39±0.28	-7.48±0.35	-7.73±0.04	-6.14±0.12	-8.32±0.09	-7.97±0.23
**15**	MOPr-DTC	-8.46±0.09	-9.06±0.3	-7.82±0.12	-6.28±0.25	-7.44±0.21	-7.62±0.04	-6.32±0.11	-8.45±0.06	-8.01±0.14
**16**	MOPr-DTC	-8.95±0.06	-9.02±0.07	-7.89±0.08	-6.10±0.18	-7.66±0.30	-7.58±0.10	-6.23±0.13	-8.33±0.08	-8.26±0.22
**17**	MOPe-DTC	-8.61±0.12	-9.20±0.14	-7.79±0.04	-6.69±0.18	-7.62±0.29	-7.87±0.05	-6.28±0.18	-8.02±0.03	-7.97±0.25
**18**	MOPe-DTC	-8.99±0.05	-9.52±0.02	-8.12±0.07	-6.62±0.39	-7.66±0.18	-7.19±0.14	-6.11±0.21	-8.60±0.02	-8.45±0.23
**19**	MOPe-DTC	-9.14±0.12	-7.42±1.19	-8.17±0.06	-6.76±0.38	-7.76±0.24	-7.46±0.17	-6.25±0.10	-8.78±0.12	-8.52±0.33
**20**	MOPe-DTC	-9.20±0.17	-9.28±0.02	-7.99±0.07	-6.61±0.25	-7.74±0.35	-7.42±0.29	-6.32±0.16	-8.55±0.25	-8.62±0.18
**21**	ODP-DTC	**-10.91±0.17**	**-10.91±0.06**	**-9.59±0.06**	-7.04±0.10	-7.85±0.50	-8.64±0.06	-7.25±0.34	4.89±1.71	-11.32±0.20
**22**	ODP-DTC	-10.84±0.12	-10.78±0.04	**-9.21±0.13**	**-7.15±0.15**	-8.14±0.64	**-9.19±0.22**	-7.11±0.23	4.23±1.55	-11.50±0.19
**23**	ODP-DTC	**-10.91±0.03**	**-10.90±0.07**	-9.13±0.06	-6.96±0.29	**-8.41±0.54**	-8.18±0.22	-7.19±0.25	-5.27±8.17	-11.52±0.16
**24**	ODP-DTC	-10.67±0.24	-10.46±0.07	-9.09±0.13	-6.90±0.51	**-8.53±0.53**	-7.25±0.23	-7.25±0.32	-9.15±0.05	**-13.25±0.19**
**25**	IST	-8.35±0.04	-8.79±0.02	-8.66±0.02	-6.91±0.50	-7.88±0.31	-8.47±0.02	-6.92±0.02	-8.32±0.02	-8.81±0.22
**26**	Brassinin	-6.57±0.08	-6.77±0.12	-6.76±0.06	-5.77±0.11	-6.62±0.07	-6.81±0.20	-5.68±0.15	-6.54±0.14	-6.81±0.17

*alkyl 2-aminoesters (**AAE**), *N*,*N*-dialkylthioureas (**NNDATU**), 2-cyanoethyl *N*-alkyldithiocarbamates (**CE-DTC**), 3-methoxy-3-oxopropyl *N*-alkyldithiocarbamate (**MOPr-DTC**), 2-methyl-4-oxopentan-3-yl *N*-alkyldithiocarbamate (**MOPe-DTC**), 2-oxo-1,3-diphenylpropyl *N*-alkyldithiocarbamates (**ODP-DTC**), 4-[(1*H*-indol-3-yl)-methylene]-2-sulfanylidene-1,3-thiazolidin-5-one (**IST**).

Table S3. Values for the Pearson correlation of the enzymes set.

ID	Mean VS	SD*																								
E1	-5.1	0.50	1.00																							
E2	**-8.4**	1.00	0.104	1.00																						
E3	-8.2	1.00	0.059	**0.879**	1.00																					
E4	-5.0	0.31	0.366	0.621	0.580	1.00																				
E5	-7.7	0.57	0.030	0.765	0.768	0.452	1.00																			
E6	-7.4	0.92	0.103	0.758	0.792	0.555	0.755	1.00																		
E7	-7.6	0.67	0.086	**0.817**	0.758	0.556	0.533	0.625	1.00																	
E8	-7.3	0.96	0.041	**0.855**	**0.820**	0.514	**0.817**	0.649	0.693	1.00																
E9	-5.4	0.34	0.292	0.514	0.470	0.618	0.341	0.474	0.426	0399	1.00															
E10	-6.1	0.54	0.230	0.498	0.385	0.375	0.434	0.366	0.338	0.381	0.364	1.00														
E11	-7.3	0.59	0.052	**0.822**	**0.810**	0.496	0.799	0.766	0.685	**0.818**	0.334	0.352	1.00													
E12	-6.7	0.47	0.207	0.577	0.566	0.547	0.389	0.404	0.513	0.494	0.373	0.328	0.495	1.00												
E13	-5.6	0.49	0.142	0.675	0.649	0.551	0.487	0.629	0.679	0.574	0.437	0399	0.644	0.504	1.00											
E14	-2.4	2.40	-0.054	-0.628	-0.621	-0.375	-0.625	-0.610	-0.479	-0.695	-0.229	-0.315	-0.694	-0.331	-0.527	1.00										
E15	-7.6	0.69	-0.007	0.785	0.759	0.513	0.767	0.719	0.626	0.745	0.314	0.382	0.734	0.523	0.531	-0.543	1.00									
E16	-6.7	0.47	0.269	0.604	0.578	0.556	0.409	0.428	0.532	0.485	0.52	0.346	0.499	0.695	0.520	-0.302	0.521	1.00								
E17	**-8.7**	2.20	0.015	0.559	0.555	0.393	0.514	0.560	0.483	0.548	0.145	0.214	0.661	0.428	0.38	-0.519	0.584	0.404	1.00							
E18	**-9.3**	1.19	-0.045	0.725	0.779	0.380	**0.807**	0.747	0.542	0.760	0.335	0.242	0.729	0.398	0.467	-0.623	0.738	0.395	0.787	1.00						
E19	-8.3	0.77	-0.623	0.790	**0.804**	0.441	**0.833**	0.785	0.619	0.719	0.328	0.378	0.762	0.478	0.519	-0.503	0.786	0.499	0.786	**0.812**	1.00					
E20	-6.6	0.53	0.091	0.261	0.125	0.275	0.057	0.088	0.524	0.194	0.168	0.152	0.110	0.095	0.280	-0.049	0.123	0.111	0.109	-0.255	0.036	1.00				
E21	-6.2	3.00	0.252	-0.219	-0.304	0.241	-0.139	-0.064	-0.313	-0.204	0.093	0.094	-0.193	-0.096	-0.120	0.111	-0.117	-0.112	0.199	-0.309	-0.273	0.041	1.00			
E22	-7.8	0.48	0.227	0.461	**0.888**	0.406	0.478	0.481	0.397	0.368	0.355	0.356	0.383	0.280	0.365	-0.319	0.326	0.298	0.026	0.423	0.398	0.143	-0.242	1.00		
E23	-6.6	0.50	0.080	0.719	0.727	0.451	0.529	0.559	0.708	0.615	0.436	0.324	0.572	0.471	0.574	-0.417	0.531	0.494	0.131	0.543	0.601	0.244	-0.489	0.589	1.00	
E24	-7.2	4.70	-0.164	-0.348	-0.260	-0.339	-0.380	-0.305	-0.213	-0.302	-0.328	-0.432	-0.234	-0.107	-0.236	0.245	-0.205	-0.077	-0.168	-0.214	-0.223	-0.181	-0.222	-0.453	-0.224	1.00
E25	**-9.6**	4.40	-0.055	0.455	0.412	0.202	0.439	0.385	0.422	0.402	0.121	0.125	0.420	0.218	0.249	0.249	0.459	0.255	0.288	0.432	0.531	0.057	-0.225	0.082	0.310	-0.053
			E1	E2	E3	E4	E5	E6	E7	E8	E9	E10	E11	E12	E13	E14	E15	E16	E17	E18	E19	E20	E21	E22	E23	E24

*mean affinity, SD (Standard Deviation).

Table S4. Antifungal activity against *Fusarium oxysporum* through mycelial growth inhibition by microscale amended-medium assay

Compound	Type	R[a]	IC_{50} (mM)	pIC_{50}(exp)[b]	pIC_{50}(pred)[c]
**1**	AAE	Me	3.10	2.51	2.37
**2**	AAE	Et	50.00	1.30	1.63
**3**	AAE	iPr	7.90	2.10	2.22
**4**	AAE	Bu	0.76	3.12	2.88
**5**	NNDATU	Me	**0.49**	3.31	3.13
**6**	NNDATU	Et	**0.76**	3.12	2.98
**7**	NNDATU	iPr	1.50	2.82	2.88
**8**	NNDATU	Bu	1.10	2.96	3.06
**9**	CE-DTC	Me	2.50	2.60	2.84
**10**	CE-DTC	Et	**0.16**	3.80	3.55
**11**	CE-DTC	iPr	2.56	2.59	2.71
**12**	CE-DTC	Bu	1.85	2.73	2.91
**13**	MOPr-DTC	Me	2.10	2.68	2.77
**14**	MOPr-DTC	Et	2.75	2.56	2.44
**15**	MOPr-DTC	iPr	2.45	2.61	2.66
**16**	MOPr-DTC	Bu	1.70	2.77	2.96
**17**	MOPe-DTC	Me	1.80	2.74	2.85
**18**	MOPe-DTC	Et	1.70	2.77	2.87
**19**	MOPe-DTC	iPr	2.44	2.61	3.01
**20**	MOPe-DTC	Bu	3.05	2.52	2.65
**21**	ODP-DTC	Me	2.50	2.60	2.75
**22**	ODP-DTC	Et	1.85	2.73	3.01
**23**	ODP-DTC	iPr	1.23	2.91	2.84
**24**	ODP-DTC	Bu	**0.44**	3.36	3.15
**25**	IST	-	1.80	2.74	2.53

[a]substitution at ester moiety; [b]pIC_{50}(exp) = -log(IC_{50}(exp) in M); [c]predicted from CoMFA model.

Table S5. Recorded interactions according to the best-docked pose of test phytoalexin analogs

Enzyme	Ligand	Residues	Interaction Type	Interacting moiety	Figure
E1	**3**	Lys5, Asn69, Asp4, Ile71, Thr2, Ser73, Gln42, Arg41	Van der Waals, Pi-sigma	dihydrochalcone, 2-propyl ester	S3.a
E2	**22**	Tyr177, Glu197, Asp173, Ser345	Van der Waals, pi-anion, Pi-cation, H-Bond	dihydrochalcone, ethyl ester, indole	S3.b
E3	**7**	Glu202, Trp356, Gln175, Asp173, Tyr171, Arg106, Trp347	H-Bond, Pi-cation	indole, sulfur, 2-propyl ester	S3.c
E4	**24**	Trp347, Arg406, Glu202, Tyr145, Gln175, Asp199, Ala174	Van der Waals, pi-anion-cation Pi, Pi-sulfide, H-Bond	dihydrochalcone, sulfur, t-butyl ester, indole	S3.e
E5	**21**	Leu373, His165, Ile28, Ala300	Pi-sigma, pi-cation, H-Bond	dihydrochalcone, sulfur, methyl ester	S3.f
E6	**25**	Gly186, Leu187, Arg182, Met221	Pi-sigma, pi-cation, H-Bond	thiazolidinone ring sulfur, indole	S3.g
E7	**7**	Lys236, Trp348, Ser343, Gln175, Ser349	Pi-sigma, pi-cation, H-Bond	indole, thiourea, methyl ester	S3.h
E8	**24**	Glu233, Leu187, Arg304, Asn185	Alkyl, Pi-sigma Pi-cation	dihydrochalcone, aromatic ring, indole	S3.i
E9	**3**	Leu182, Lys151, Asn84	Pi-sulfide Van der Waals	dihydrochalcone, indole, 2-propyl ester	S3.j
E10	**6**	Ser120, Leu182, Glu44, Val184, Leu189, His188, Val184	H-Bond	thiourea (sulfur and nitrogen), indol	S3.k
E11	**6**	Leu189, Thr43, Ser120, Tyr119, His188	Van der Waals, pi-anion, Pi-cation, sulfur, H-Bond	indole, thiourea (sulfur)	S3.l
E12	**25**	Ala517, Thr518	alkyl	Indole, thiazolidinone	S3.m
E13	**22**	His282, Gln504, Glu500, Val280, Ile281, Lys283	Pi-sigma, pi-cation, H-Bond	dihydrochalcone, indole, ethyl ester	S3.n
E14	**24**	Met 218, Lys 513, Ala517	Pi-sigma Pi-cation, H-Bond, Pi-alkyl	indole, butyl ester	S3.n
E15	**25**	Val162, Trp380, Ala292, Lys382, Asp262	Pi-sigma Pi-alkyl, H-Bond	indole,thiazolidinone	S3.o
E16	**22**	Leu99, Thr139, Phe273, Trp142, Glu397, Met379	H-Bond, Pi-sigma Pi-alkyl, Pi-sulfide	dihydrochalcone, ethyl ester, indole	S3.p
E17	**5**	Gly404, Trp142, Phe401,273, Val377, Ser134, Arg304	H-Bond, Pi-alkyl, alkyl, Pi-sigma	thiourea, indol, methyl	S3.q
E18	**5**	Met379, Leu400, Ala140, Glu135, Thr139, Gly138, His133, Ile310	H-Bond, Pi-cation	indole, methyl, thiourea (nitrogen)	S3.r
E19	**1**	Ans241, Ala242, Thr175, Val245	H-Bond	indole, methyl	S3.s
E20	**1**	Lys179, Leu178, Asn403, Arg222, Asp408	H-Bond, Pi-alkyl, alkyl, Pi-sigma	dihydrochalcone phenyl	S3.u
E22	**22**	Tyr139, Lys188, Arg175, Asp208, Met367, Ala77	H-Bond, Pi-alkyl, alkyl, Pi-sigma	dihydrochalcone, aromatic ring, methyl ester	
E23	**7**	Ile304, Ile304, Lys271, Asn273, Pro97, Leu128, Hid274, Arg277, Gly185	H-Bond, alkyl Pi, Pi-sigma	indole, methyl	S3.v

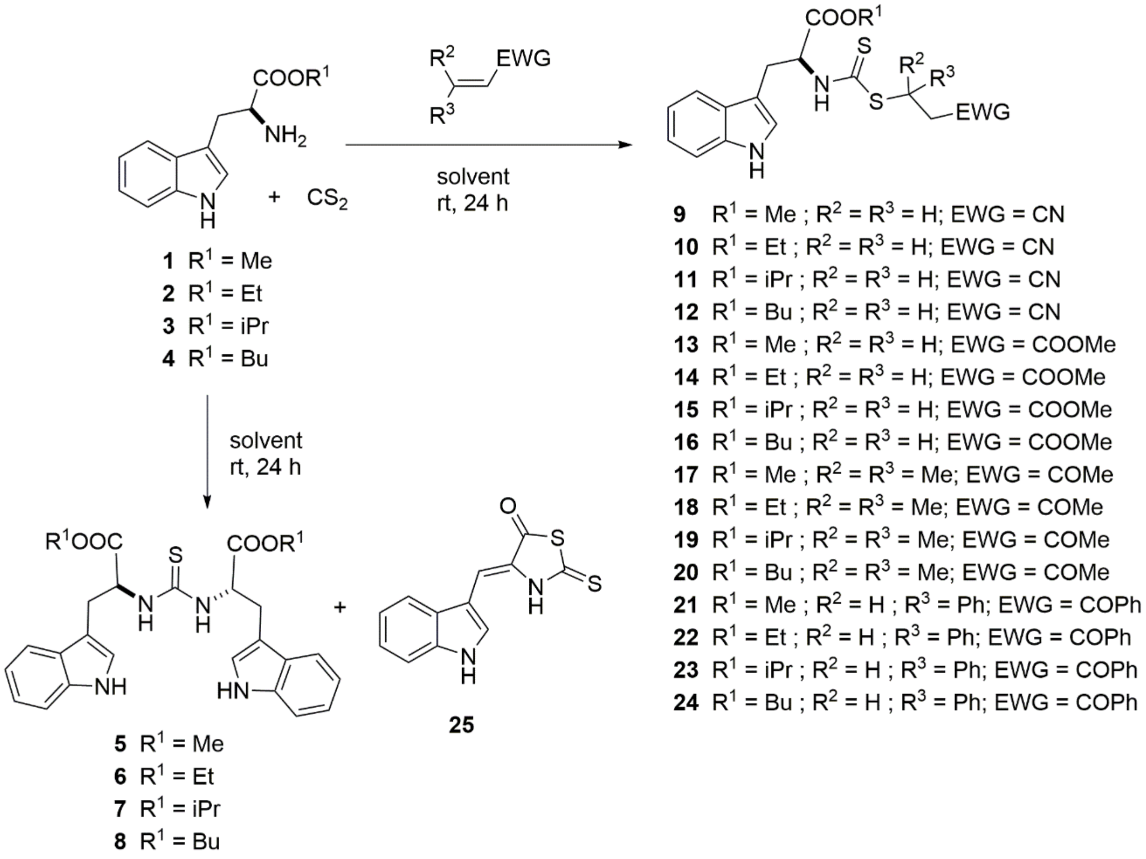

Figure S1. Reaction route for obtaining the test indole-containing phytoalexin analogues.

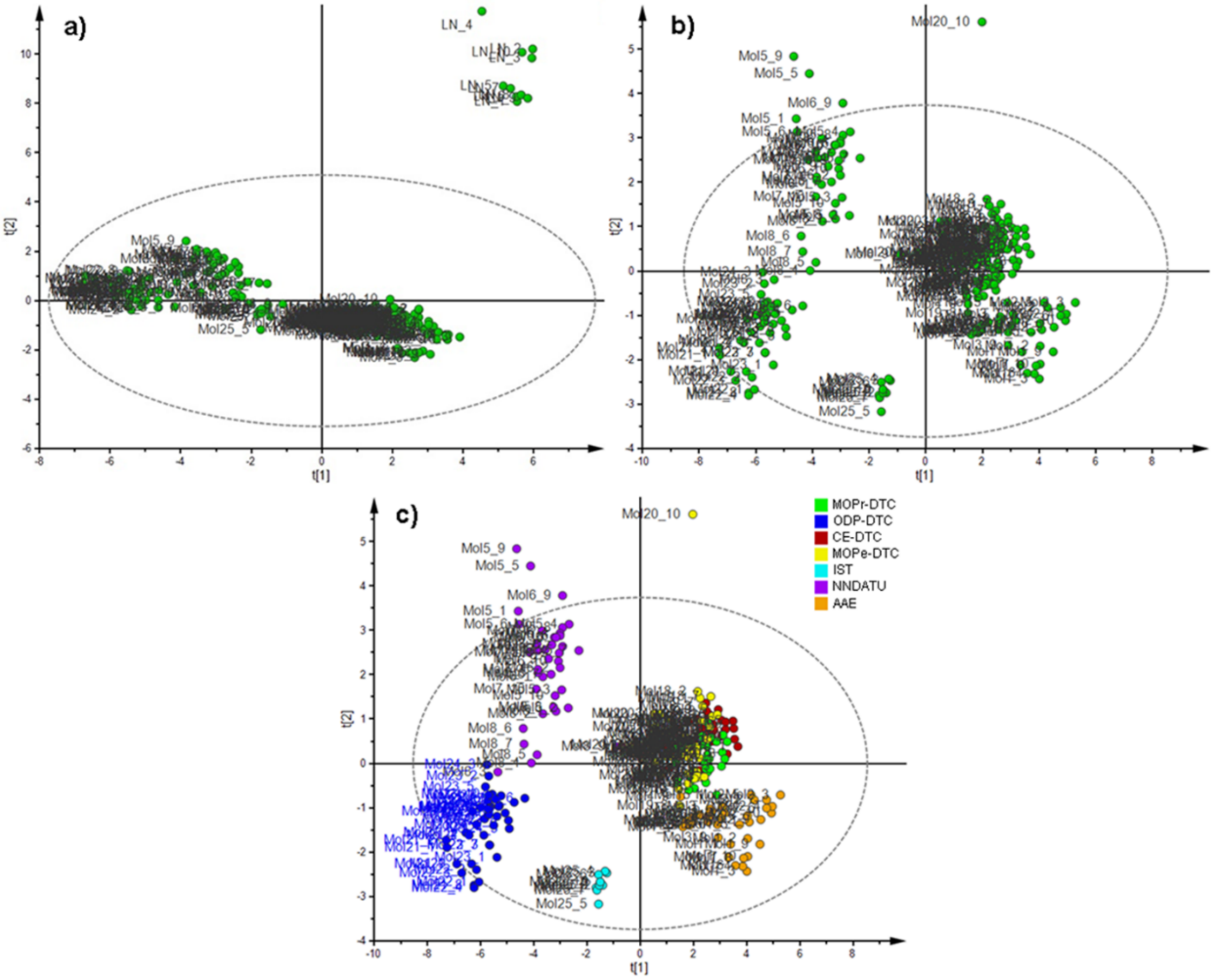

Figure S2. PCA-derived score plots from the unsupervised multivariate analysis from docking scores dataset for the test indole-containing phytoalexins analogs. **a**) involving natural ligands, **b**) excluding natural ligands, **c**) coloration according compound type: alkyl 2-aminoesters (**AAE**), *N*,*N*-dialkylthioureas (**NNDATU**), 2-cyanoethyl *N*-alkyldithiocarbamates (**CE-DTC**), 3-methoxy-3-oxopropyl *N*-alkyldithiocarbamate (**MOPr-DTC**), 2-methyl-4-oxopentan-3-yl *N*-alkyldithiocarbamate (**MOPe-DTC**), 2-oxo-1,3-diphenylpropyl *N*-alkyldithiocarbamates (**ODP-DTC**), 4-[(1*H*-indol-3-yl)-methylene]-2-sulfanylidene-1,3-thiazolidin-5-one (**IST**).

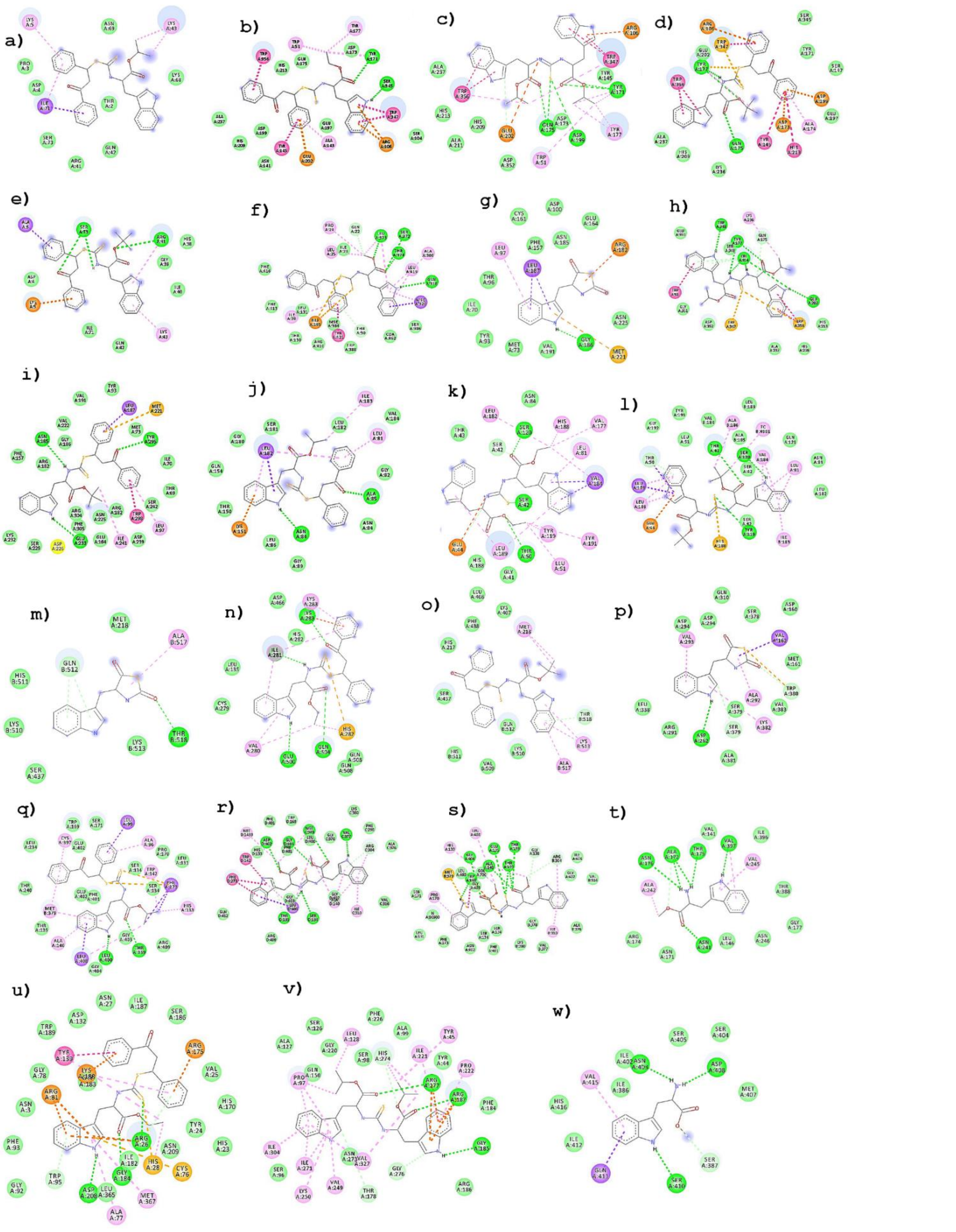

Figure S3: 2D-residual interactions for the test indole-containing phytoalexin analogues. (a) E1-mol23; (b) E2-mol22; (c) E3-mol7; (d) E3-mol24; (e) E4-mol24; (f) E5-mol21; (g) E6-mol25, (h) E7-mol7; (i) E8-mol24; (j) E9-mol23; (k) E10-mol6; (l) E11-mol6; (m) E12-mol25; (n) E13-mol22; (n˜) E14-mol24; (o) E15-mol25; (p) E16-mol22; (q) E17-mol5; (r) E18-mol5; (s) E19-mol1; (t) E20-mol1; (u) E22-mol22; (v) E23-mol7; (w) E24-mol1.
